# Multidimensional Assessment of Neurological Adverse Reactions Related to PD‐1 Inhibitors: A Real‐World Pharmacovigilance Study

**DOI:** 10.1002/cns.70734

**Published:** 2026-01-05

**Authors:** Xiaofeng Hu, Xiaoli Wang, Bufu Tang, Dehuan Zhang, Rongbing Cai, Hui Jiang, Yaling Lin, Yiheng Song, Yiou Wang, Hairuo Huang, Dandan Guo, Xubin Sun, Hongjie Fan

**Affiliations:** ^1^ Department of Oncology Guangyuan Central Hospital Guangyuan Sichuan China; ^2^ Department of Obstetrics and Gynecology, Tongji Hospital, Tongji Medical College Huazhong University of Science and Technology Wuhan P.R. China; ^3^ Department of Interventional Radiology, Zhongshan Hospital Fudan University Shanghai China; ^4^ Dalian Medical University Dalian P.R. China; ^5^ The Fourth Clinical College of China Medical University Shenyang China; ^6^ Department of Radiology, Union Hospital, Tongji Medical College Huazhong University of Science and Technology Wuhan China

**Keywords:** adverse events, FAERS, immunotherapy, neurological adverse events, PD‐1 inhibitor, pharmacovigilance

## Abstract

**Background:**

PD‐1 inhibitors have revolutionized cancer immunotherapy but present significant neurological safety concerns. While clinical trials have documented neurological adverse events (nAEs), a comprehensive understanding of their patterns and risk factors remains limited. This study systematically analyzed a decade of FAERS data to investigate PD‐1 inhibitor–associated neurotoxicities.

**Methods:**

Using FAERS data (2014–2024), we conducted disproportionality analyses (ROR, IC, PRR) to assess PD‐1 inhibitor–nAE associations. Risk factors were evaluated using logistic regression; timing analyses used log‐rank and Mann–Whitney *U* tests, while group comparisons used Chi‐square tests.

**Results:**

Among 115,000 PD‐1 inhibitor–associated adverse events, 7968 (6.93%) involved nAEs, showing an increasing trend from 4.96% (Q4 2014) to 7.67% (Q1‐Q2 2024). PD‐1 inhibitors showed significant nAE signals (ROR: 1.21, 95% CI: 1.18–1.23), with cemiplimab showing the strongest association (ROR: 1.38, 95% CI: 1.18–1.62). The most common nAEs were dizziness (*N* = 942, 10.3%), encephalitis (*N* = 435, 4.8%), and cerebrovascular accident (*N* = 451, 4.9%). Risk factors included age > 65 years (OR: 1.10), female sex (OR: 1.04), skin cancer (OR: 1.36), and nervous system cancers (OR: 1.44). The median onset time was 34 days (IQR: 12–104), with 63.8% occurring within 2 months and 59% resulting in severe outcomes.

**Conclusion:**

This study reveals a spectrum of PD‐1 inhibitor–related neurological toxicities, mainly involving central nervous system dysfunction, providing important insights into risk patterns and timing characteristics. These findings support improved clinical monitoring practices and inform the development of personalized patient care strategies.

## Graphical abstract

This pharmacovigilance study systematically analyzed PD‐1 inhibitor–associated neurological adverse events using FAERS data from 2014 to 2024. Central nervous system toxicities, including encephalitis and brainstem encephalitis, were most prominent. Older age, female sex, specific cancer types, and combination therapies increased the risk, with most events occurring within 2 months and over half being severe.

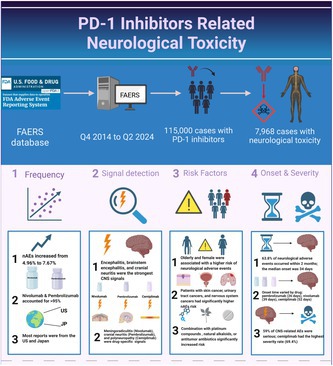

**Abbreviations**
AEs: adverse events
CNS: central nervous system
FAERS: FDA Adverse Event Reporting System
FDA: Food and Drug Administration
nAEs: neurological adverse events
PD‐1: programmed cell death protein 1
PT: preferred term
SOC: System Organ Classification

## 1 Introduction

Cancer immunotherapy, particularly immune checkpoint inhibitors (ICIs) such as programmed cell death protein 1 (PD‐1) inhibitors, has significantly advanced cancer treatment by enhancing the immune system's ability to target and eliminate malignant cells [1]. PD‐1 inhibitors function by blocking PD‐1 on T cells from binding to PD‐L1/PD‐L2, which are frequently overexpressed on tumor cells. This blockade prevents T‐cell exhaustion and restores antitumor immune responses by reactivating T‐cell proliferation, cytokine production, and cytotoxic function, thereby enhancing tumor cell recognition and elimination [2]. Currently, six PD‐1 inhibitors, including nivolumab, pembrolizumab, cemiplimab, dostarlimab, toripalimab, and tislelizumab, have received United States Food and Drug Administration (U.S. FDA) approval for cancer treatment [3, 4, 5, 6, 7, 8]. These PD‐1 inhibitors have demonstrated significant therapeutic efficacy in multiple malignancies, including melanoma, non‐small‐cell lung cancer (NSCLC), and hepatocellular carcinoma (HCC), supported by robust clinical evidence and extensive real‐world experience [9, 10].

Despite their remarkable clinical benefits, PD‐1 inhibitors can lead to immune‐related adverse events (irAEs) due to enhanced immune system activation, presenting significant challenges in clinical management. These irAEs commonly affect the endocrine, pulmonary, gastrointestinal, and hepatic systems. Less frequent but potentially life‐threatening complications include neurological, cardiovascular, and severe pulmonary adverse events [11]. Neurological adverse events (nAEs), though relatively uncommon, represent a particularly concerning subset of irAEs. These neurological manifestations span from quality‐of‐life‐impacting symptoms (such as visual disturbances, neurogenic bladder, and headaches) to severe, potentially fatal conditions (including myasthenia gravis, encephalitis, and seizures) [12]. These symptoms may be more common and severe in patients with different clinical characteristics and indications, requiring immediate intervention in severe cases. Additionally, while the onset of symptoms is generally concentrated in the early stages of treatment, variations in timing can occur based on individual patient characteristics [13]. Therefore, implementing targeted management strategies for high‐risk patients, coupled with a comprehensive understanding of onset patterns across different patient subgroups, may optimize therapeutic outcomes and enhance treatment safety.

While clinical observations and reviews have documented the neurological adverse events associated with PD‐1 inhibitors, most available evidence comes from clinical trials with limited patient populations, and large‐scale, long‐term real‐world data are still needed [14, 15, 16]. Studies comparing the neurological adverse event profiles among different PD‐1 inhibitors (such as nivolumab, pembrolizumab, and cemiplimab) remain limited. Additionally, while factors such as age, gender, therapeutic indications, and concomitant medications may influence the occurrence of these neurological adverse events, research on these potential risk factors is still insufficient. These knowledge gaps affect evidence‐based clinical decision‐making in risk prediction and management of PD‐1 inhibitor–related neurological adverse events.

Given these knowledge gaps, a comprehensive pharmacovigilance study using large real‐world data is needed. The major purpose of this study was to analyze data from the FAERS database ranging from 2014 to 2024 to explore the relationship between neurological adverse events and PD‐1 inhibitors and examine their occurrence trends, clinical characteristics, and potential risk factors. Using pharmacovigilance signal detection methods, we investigated the associations between specific PD‐1 inhibitors and neurological adverse events, with additional analysis of patient‐related and treatment‐related factors influencing event occurrence. This systematic analysis sought to establish evidence‐based guidance for clinicians on risk assessment and management of PD‐1 inhibitor–induced neurological adverse events for malignancy therapy. These findings may enhance pharmacovigilance capabilities and inform clinical decision‐making, potentially contributing to improved patient safety outcomes.

## 2 Method

### 2.1 Data Source

We conducted a retrospective pharmacovigilance analysis using the FAERS database, a globally recognized, publicly accessible repository for adverse event (AE) reports, with a focus on PD‐1 inhibitors. The FAERS database consolidates voluntary reports from various sources, including healthcare professionals, patients, pharmacists, and pharmaceutical companies, supporting the FDA's postmarket surveillance of drugs and biologics. The FAERS quarterly file package includes seven data files: DEMO (patient demographics and administrative information), DRUG (drug information), REAC (adverse event codes), OUTC (patient outcomes), RPSR (report sources), THER (therapy start and end dates), and INDI (indications for drug use). These files provide comprehensive information on AEs, linked to PRIMARYID, CASEID, and drug_seq [17]. The database is updated quarterly, with data available at https://fis.fda.gov/extensions/FPD‐QDE‐FAERS/FPD‐QDE‐FAERS.html.

AEs in the FAERS database were classified using Preferred Term (PT) codes from the Medical Dictionary for Regulatory Activities (MedDRA), a standardized terminology system for clinical validation in the biopharmaceutical industry. MedDRA standardizes data across the full regulatory procedure, from preapproval and postapproval phases. It includes five hierarchical levels: System Organ Class (SOC), High‐Level Group Term (HLGT), High‐Level Term (HLT), Preferred Term (PT), and Lowest Level Term (LLT). The SOC categorizes terms by body systems, while HLGT represents broader groupings of medical conditions, and HLT provides more specific terms. PT is the core unit, detailing specific medical events, and LLT offers even more granular descriptions. MedDRA's multiaxial structure allows a single PT to be linked to multiple SOCs, although each PT has one primary SOC.

### 2.2 Procedures

**FIGURE 1:**
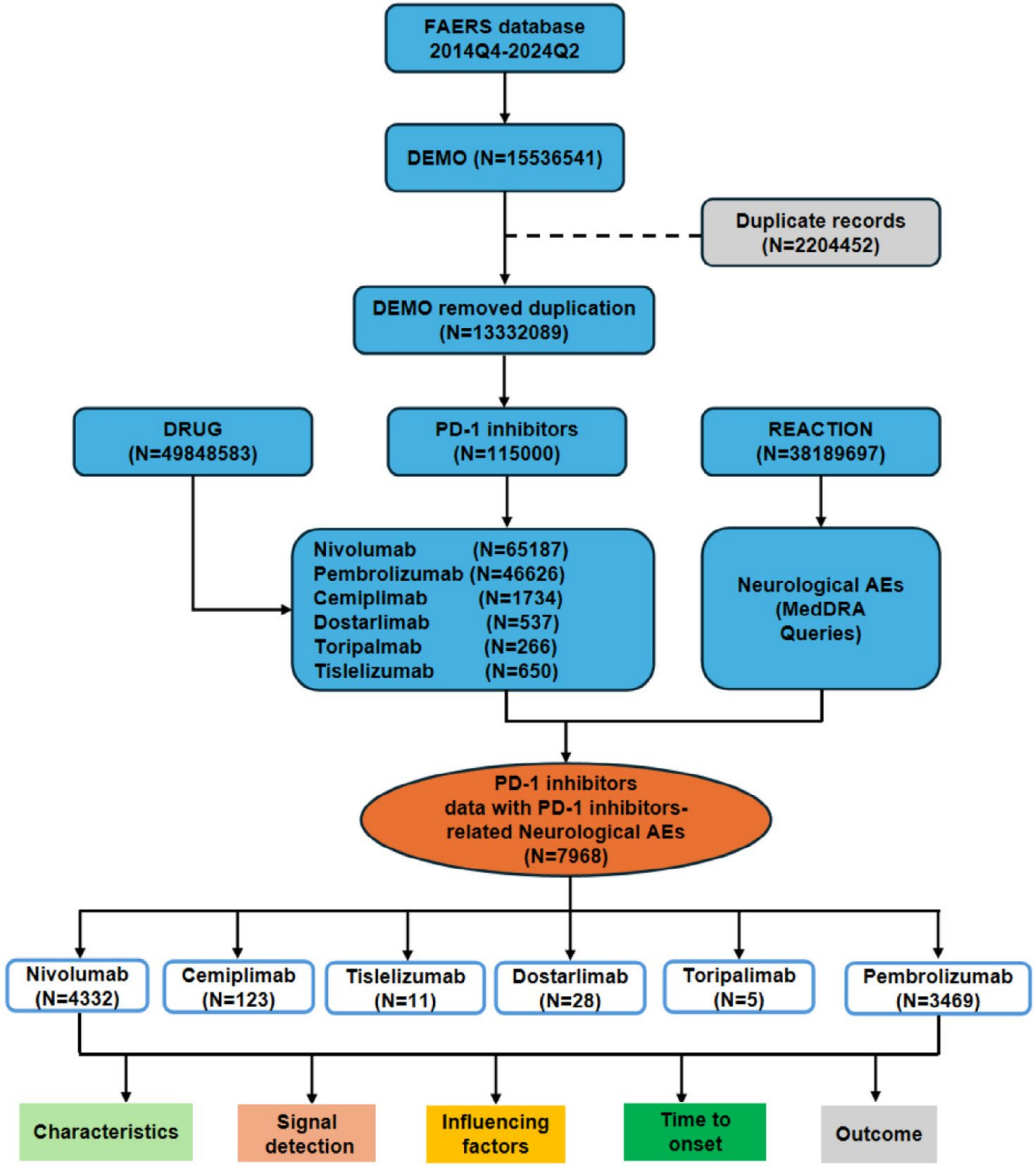
Flowchart illustrates the data processing and analysis workflow of this study.

The present research analyzed data using the openly accessible FAERS dataset, spanning the years 2014–2024. The PD‐1 inhibitors investigated include nivolumab, pembrolizumab, cemiplimab, toripalimab, tislelizumab, and dostarlimab. Due to the lack of standardized drug nomenclature in the FAERS database, instances involving PD‐1 inhibitors were identified using both brand and generic names. Neurological adverse reactions were screened using the System Organ Class (SOC) “Nervous System Disorders” and the primary SOC as “yes,” including related conditions such as myasthenia gravis and neurogenic bladder. AEs in the FAERS database are classified using MedDRA terminology, with drugs categorized as PS (primary suspect), SS (second suspect), C (concomitant), and I (interacting) [18, 19, 20]. For accuracy, we focused on cases where the drug's role was marked as “PS.” Data accuracy was ensured by following the FDA's methodology for eliminating duplicate reports. We filtered duplicate cases by keeping only the latest FDA‐tracked records. When both case numbers and FDA Document Tracking dates matched, the report possessing the higher Individual Safety Report number was retained. Duplicates identified by matching fields such as age, sex, country, event date, adverse event, drug, and indication were excluded, ensuring only the most recent version remained in the dataset. The complete procedure is depicted in Figure 1.

### 2.3 Disproportionality Analysis

In this investigation, we utilized disproportionality analysis, also known as case versus noncase analysis, a popular tool for signal discovery in pharmacovigilance [21]. We employed three widely used disproportionality analysis methods—the Reporting Odds Ratio (ROR), the Information Component (IC), and the Proportional Reporting Ratio (PRR)—to evaluate the correlation between PD‐1 inhibitors and neurological adverse reactions [22, 23, 24, 25]. The following formulas were used to calculate the ROR, IC, PRR, and their respective 95% confidence intervals (CIs).

$$\mathrm{ROR}=\frac{a\times d}{b\times c}\;95\%\mathrm{CI}=\exp\left(\text{In}\left(\mathrm{ROR}\right)\pm 1.96\times \sqrt{\frac{1}{a}+\frac{1}{b}+\frac{1}{c}+\frac{1}{d}}\right)$$

$$\begin{array}{cc} \mathrm{IC}=\log\left(\frac{a}{b}\times \frac{c}{d}\right) & 95\%\mathrm{CI}=\mathrm{IC}\pm 1.96\times \sqrt{\frac{1}{a}+\frac{1}{b}+\frac{1}{c}+\frac{1}{d}} \end{array}$$

$$\begin{array}{cc} \mathrm{PRR}=\frac{\frac{a}{a+b}}{\frac{c}{c+d}} & 95\%\mathrm{CI}=\exp\left(\ln\left(\mathrm{PRR}\right)\pm 1.96\times \sqrt{\frac{1}{a}+\frac{1}{b}+\frac{1}{c}+\frac{1}{d}}\right) \end{array}$$

In this analysis, “*a*” denotes the number of nAEs reported with PD‐1 inhibitors, “*b*” denotes the number of other AEs reported with PD‐1 inhibitors, “*c*” denotes the number of nAEs associated with other drugs, and “*d*” denotes the number of other AEs reported with other drugs. For signals related to PD‐1 inhibitors and overall nAEs, an ROR with a lower 95% CI greater than 1 is considered valid. However, for individual nAEs, the criteria are more stringent: the signal is considered significantly associated with PD‐1 inhibitors if there are three or more reports, the lower bound of the 95% CI for the Information Component (IC) is greater than zero, and the lower bound of the 95% CI for the ROR exceeds one. Neurological disorder Preferred Terms (PTs) that meet these criteria were classified as PD‐1 inhibitor–related nAEs [26].

### 2.4 Descriptive Analysis

A descriptive analysis was conducted on the clinical characteristics of reports related to PD‐1 inhibitor–associated nAEs, including sex, age, weight, submitter, indication, outcome, time to onset, concomitant medications, co‐reported AEs, PD‐1 inhibitor type, and country. Outcomes were classified as severe (including death, near death, and hospitalization) or nonsevere (all other outcomes). Reporters were categorized as professionals (physicians, pharmacists, and other healthcare providers) or nonprofessionals (consumers and attorneys). Time to onset was determined by subtracting the onset time of the adverse event from the start of treatment.

### 2.5 Statistical Analysis

We summarized Categorical variables using counts and proportions. The temporal patterns of onset across groups underwent analysis via Log‐rank testing and Mann–Whitney *U* procedures. Group comparisons for outcomes employed chi‐square analysis. Logistic regression analysis was conducted to assess the impact of each clinical characteristic on PD‐1 inhibitor–induced nAEs, with odds ratios (ORs) and corresponding 95% CIs calculated. Missing values were excluded from analyses rather than being imputed. Before all statistical analyses, the normality of continuous variables was assessed using the Shapiro–Wilk test for samples ≤ 50 and the Kolmogorov–Smirnov test for larger samples (*n* > 50) to enhance statistical robustness. Variables demonstrating normal (Gaussian) distribution were analyzed using parametric tests (independent samples *t*‐test, one‐way ANOVA), while those failing normality assumptions were analyzed using appropriate nonparametric alternatives (Mann–Whitney *U* test, Kruskal–Wallis test). This approach ensured optimal statistical methodology based on underlying data distribution characteristics. A *p*‐value of less than 0.05 was considered statistically significant, with all tests being two‐sided. The data analysis and processing were performed by R Studio. Data visualization was conducted with both R Studio and Python. The human anatomy heatmap was generated using the online tool available at https://smuonco.shinyapps.io/MOAHIT/. This research followed the STROBE guidelines to ensure proper reporting [27]. Temporal trend analysis was conducted using linear regression of quarterly aggregated neurological adverse event counts from 2014 to 2024. The model assessed the relationship between time (quarters) and event frequency, with statistical significance evaluated at *p* < 0.05. Model diagnostics confirmed adherence to regression assumptions.

### 2.6 Data Management and Quality Considerations

The FAERS database, as a voluntary spontaneous reporting system, has inherent limitations that should be considered when interpreting our findings. Data quality and completeness naturally vary across reports, influenced by factors such as reporter type, geographic location, and clinical context. Healthcare professionals typically provide more detailed clinical information, and reporting patterns may differ across countries and healthcare systems. Spontaneous reporting databases may reflect various reporting tendencies, with some events receiving more attention than others. These features are inherent to passive surveillance systems and inform our choice of disproportionality methods, which are designed for signal detection and hypothesis generation in pharmacovigilance research.

Missing data pose a notable challenge in FAERS analyses, with varying degrees of incompleteness across different variables. In our dataset, body weight information was missing in 70.0% of reports, time to event onset in 61.3%, and age in 25.0%. Consistent with the reporting patterns described above, the extent of incomplete data was systematically related to reporter type and geographic origin, with healthcare professionals providing more complete information than consumers. Given the nonrandom nature of missing data, we did not employ imputation methods, as these assume data are missing at random and could introduce bias when violated. Instead, we used complete‐case analysis, including only reports with complete information for all required variables in each analysis. For example, analyses examining body weight‐outcome relationships included only cases with documented weight values, while time‐to‐event analyses required complete onset time information. While this reduces sample size, it preserves validity by avoiding biases from inappropriate imputation.

### 2.7 Sensitivity Analyses and *E*‐Value Calculation

To assess the robustness of our findings to potential unmeasured confounding, we conducted sensitivity analyses using multivariable logistic regression and calculated *E*‐values. Adjusted reporting odds ratios (aRORs) and 95% confidence intervals (CIs) were calculated using two models: Model 1 adjusted for reporter type (professional vs. unprofessional), geographic region (United States vs. other countries), and concomitant medication use (monotherapy vs. polypharmacy); Model 2 additionally adjusted for age (continuous variable), sex (male vs. female), and body weight (continuous variable). Complete‐case analysis was employed for all regression models. *E*‐values were calculated for both unadjusted reporting odds ratios and adjusted reporting odds ratios to quantify the minimum strength of association on the risk ratio scale that an unmeasured confounder would need to have with both PD‐1 inhibitor exposure and neurological adverse events to fully explain away the observed associations [28]. Under the rare outcome assumption (nAE incidence < 10%), reporting odds ratios and adjusted reporting odds ratios were treated as approximations of risk ratios for *E*‐value calculation ($E-\text{value}=\mathrm{RR}+\sqrt{\left(\mathrm{RR}\times \left(\mathrm{RR}-1\right)\right)}$), where RR represents the (adjusted) reporting odds ratio or its confidence limit. *E*‐values were computed for both point estimates and the lower bounds of 95% confidence intervals, with the latter representing a more conservative assessment of robustness to unmeasured confounding [29].

## 3 Results

### 3.1 Neurological Adverse Events Among PD‐1 Inhibitor Users: FAERS Database Reports From Q4 2014 to Q2 2024

**FIGURE 2:**
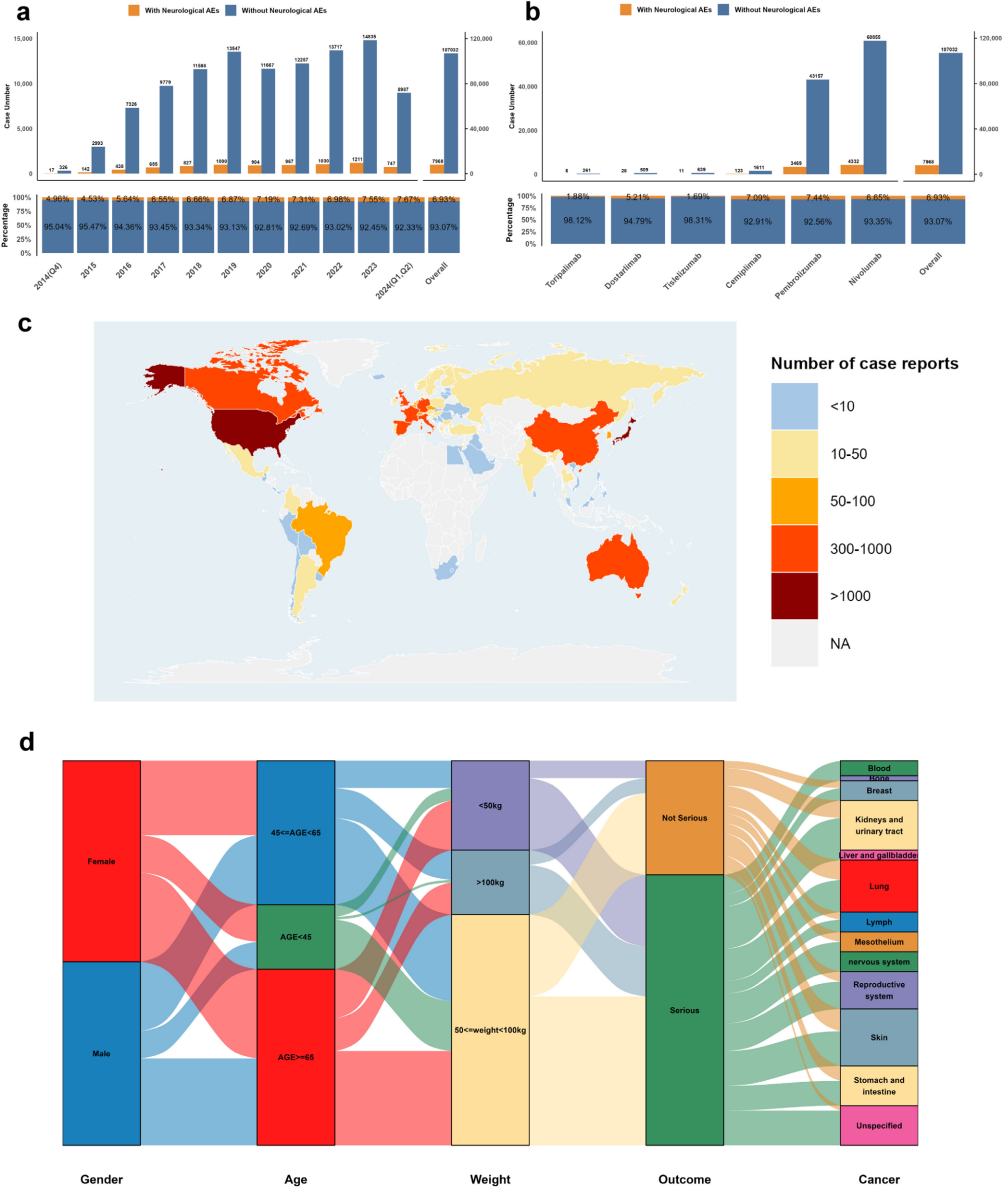
Temporal, drug‐specific, geographic, and demographic distribution of neurological adverse events (nAEs) associated with PD‐1 inhibitors from Q4 2014 to Q2 2024. (a) Upper panel: Stacked bar chart of neurological (orange) and non‐neurological (blue) adverse events by year from Q4 2014 to Q2 2024. Lower panel: Annual proportions of nAEs vs. non‐nAEs. (b) Upper panel: Stacked bar chart of neurological (orange) and non‐neurological (blue) adverse events by PD‐1 inhibitor type. Lower panel: Proportions of nAEs vs. non‐nAEs for each inhibitor. (c) World map showing the global distribution of neurological adverse event reports. Color represents case frequency ranges (< 10 to > 1000 cases). (d) Sankey diagram of patient characteristics including gender, age groups, weight categories, outcomes (serious/nonserious), and cancer types. Flow width indicates patient numbers.

Among 115,000 PD‐1 inhibitor–associated AEs identified in the FAERS database (Q4 2014–Q2 2024), 7968 cases (6.93%) involved neurological adverse events (NAEs). The proportion of NAEs showed an increasing trend from 4.96% (Q4 2014) to 7.67% (Q1–Q2 2024), with annual cases in 2021–2023 reaching 1211, 1030, and 967 respectively, representing a substantial elevation compared to 2015–2020 (Figure 2a). Within all PD‐1 inhibitors, nivolumab (*n* = 4332) and pembrolizumab (*n* = 3469) dominated the NAE reports, together accounting for 97.9% of all cases, while recently approved agents—toripalimab (*N* = 5), tislelizumab (*N* = 11), and dostarlimab (*N* = 28)—showed markedly lower frequencies, likely due to their limited postmarketing exposure (Figure 2b). The United States (*N* = 2655; 33.3%) and Japan (*N* = 1882; 23.6%) contributed the highest numbers of NAE cases, followed by France (*N* = 672; 8.4%), Germany (*N* = 429; 5.4%), Britain (*N* = 199; 2.5%), and China (*N* = 191; 2.4%) (Figure 2c). Overall, the proportions and growth trends of neurological adverse reactions caused by PD‐1 inhibitors of different types and in different locations in various years suggest that this may be an indispensable aspect of PD‐1 inhibitor therapy.

### 3.2 Descriptive Analysis of PD‐1 Inhibitor–Induced Neurological Adverse Events

The clinical characteristics of individuals with PD‐1 inhibitor–induced nAEs are displayed in the Table 1. Among all adverse events reported with PD‐1 inhibitors, male patients (*N* = 4307, 54.1%) significantly outnumbered female patients (*N* = 3052, 38.3%). In the pembrolizumab treatment group, the gender distribution was balanced between male (*N* = 1682, 48.5%) and female patients (*N* = 1634, 47.1%). The patient cohort exhibited a median age of 67 years (IQR 58–74). In both the general population and in specific PD‐1 inhibitor groups, the majority of patients were aged ≥ 65 (*N* = 3462, 43.4%). Patients had a median weight of 70 kg (IQR 58–83), with the majority of patients (*N* = 1947, 24.4%) within the 50–100 kg range. Most cases were reported as serious (*N* = 4703, 59%), and the predominant portion of reports originated from healthcare professionals (*N* = 4698, 59%). For combination therapy, additional platinum‐based compounds were most frequently administered (*N* = 845, 10.6%), followed by additional protein kinase inhibitors (*N* = 579, 7.3%) and additional natural alkaloids and derivatives (549, 6.9%) (Table 1). In terms of distribution by indication, lung cancer (*N* = 2165, 27.2%), skin cancer (*N* = 1639, 20.6%), and kidneys and urinary tract (*N* = 1280, 16.1%) constituted the predominant categories (Figure 4e,f). Comprehensive clinical characteristics flow patterns were illustrated in Figure 2d via a Sankey diagram.

**TABLE 1:** Clinical characteristics of Reports on nAEs Induced by PD‐1 Inhibitors in the FAERS Database (Q4 2014–Q2 2024).

Clinical characteristics	Overall (*N* = 7968)	Nivolumab (*N* = 4332)	Pembrolizumab (*N* = 3469)	Cemiplimab (*N* = 123)	Others (*N* = 44)
Gender					
Male	4307 (54.1%)	2580 (59.6%)	1682 (48.5%)	44 (35.8%)	1 (2.3%)
Female	3052 (38.3%)	1387 (32.0%)	1634 (47.1%)	25 (20.3%)	6 (13.6%)
Missing	609 (7.6%)	365 (8.4%)	153 (4.4%)	54 (43.9)	37 (84.1%)
Age					
Median [IQR]	67 [58–74]	66 [58–73]	68 [58–75]	71 [59–78]	70 [53–73]
Mean (SD)	64 (14)	64 (13)	65 (16)	69 (12)	64 (14)
< 45	438 (5.5%)	245 (5.7%)	192 (5.5%)	1 (0.8%)	0 (0%)
45–65	2084 (26.1%)	1252 (28.9%)	815 (23.5%)	15 (12.2%)	2 (4.5%)
≥ 65	3462 (43.4%)	1896 (43.8%)	1537 (44.3%)	26 (21.1%)	3 (6.8%)
Missing	1984 (25.0%)	939 (21.6%)	925 (26.7%)	81 (65.9%)	39 (88.6%)
Weight (kg)					
Median [IQR]	70 [58–83]	71 [59–85]	68 [57–80]	63 [61–88]	68 [62–75]
Mean (SD)	72 (21)	74 (21)	71 (20)	78 (30)	70 (15)
< 50	231 (2.9%)	125 (2.9%)	105 (3.0%)	1 (0.8%)	0 (0%)
50–100	1947 (24.4%)	1163 (26.8%)	777 (22.4%)	2 (1.6%)	5 (11.4%)
≥ 100	216 (2.7%)	142 (3.3%)	73 (2.1%)	1 (0.8%)	0 (0%)
Missing	5574 (70.0%)	2902 (67.0%)	2514 (72.5%)	119 (96.8%)	39 (88.6%)
Time to onset (day)					
Median [IQR]	34 [12–104]	39 [14–112]	26 [7–84]	52 [19–112]	39 [1109]
Mean (SD)	97 (200)	104 (220)	81 (152)	123 (201)	86 (153)
Missing	4884 (61.3%)	2297 (53.0%)	2499 (72.0%)	66 (53.7%)	22 (50.0%)
Outcome					
Serious	4703 (59.0%)	2739 (63.2%)	1866 (53.8%)	75 (61.0%)	23 (52.3%)
Not serious	2647 (33.2%)	1372 (31.7%)	1226 (35.3%)	33 (26.8%)	16 (36.4%)
Missing	618 (7.8%)	221 (5.1%)	377 (10.9%)	15 (12.2%)	5 (11.4%)
Submitter					
Professional	4698 (59.0%)	2676 (61.8%)	1916 (55.2%)	85 (69.1%)	21 (47.7%)
Unprofessional	2061 (25.9%)	893 (20.6%)	1137 (32.8%)	23 (18.7%)	8 (18.2%)
Missing	1209 (15.1%)	763 (17.6%)	416 (12.0%)	15 (12.2%)	15 (34.1%)
Country					
United States	2655 (33.3%)	1365 (31.5%)	1222 (35.2%)	54 (43.9%)	14 (31.8%)
Japan	1882 (23.6%)	857 (19.8%)	1015 (29.2%)	10 (8.1%)	0 (0%)
France	672 (8.4%)	417 (9.6%)	246 (7.1%)	8 (6.5%)	1 (2.3%)
Germany	429 (5.4%)	303 (7.0%)	121 (3.5%)	2 (1.6%)	3 (6.8%)
China	191 (2.4%)	121 (2.8%)	54 (1.6%)	0 (0%)	16 (36.4%)
Britain	199 (2.5%)	114 (2.6%)	83 (2.4%)	2 (1.6%)	0 (0%)
Others	1940 (24.4%)	1155 (26.7%)	728 (21.0%)	47 (38.3%)	10 (22.7%)
Additional drug					
Platinum based compounds	845 (10.6%)	211 (4.9%)	617 (17.8%)	6 (4.9%)	11 (25.0%)
Protein kinase inhibitor	579 (7.3%)	123 (2.8%)	455 (13.1%)	1 (0.8%)	0 (0%)
Natural alkaloids and derivatives	549 (6.9%)	122 (2.8%)	414 (11.9%)	3 (2.4%)	10 (22.7%)
Metabolic antagonists	364 (4.6%)	111 (2.6%)	248 (7.1%)	3 (2.4%)	2 (4.5%)
Alkylating agents	112 (1.4%)	27 (0.6%)	85 (2.5%)	0 (0%)	0 (0%)
Antitumor antibiotics	85 (1.1%)	11 (0.3%)	74 (2.1%)	0 (0%)	0 (0%)
Hormones and antihormones	44 (0.6%)	18 (0.4%)	24 (0.7%)	2 (1.6%)	0 (0%)
Indication (Cancer)					
Lung	2165 (27.2%)	1086 (25.1%)	1052 (30.3%)	20 (16.2%)	7 (15.9%)
Skin	1639 (20.6%)	1103 (25.5%)	500 (14.4%)	36 (29.3%)	0 (0%)
Kidneys and urinary tract	1280 (16.1%)	850 (19.6%)	427 (12.3%)	0 (0%)	3 (6.8%)
Stomach and intestine	596 (7.5%)	407 (9.4%)	184 (5.3%)	4 (3.3%)	1 (2.3%)
Reproductive system	448 (5.6%)	46 (1.1%)	379 (10.9%)	7 (5.7%)	16 (57.1%)
Breast	330 (4.1%)	14 (0.3%)	315 (9.1%)	0 (0%)	1 (3.6%)
Lymph	114 (1.4%)	83 (1.9%)	28 (0.8%)	3 (2.4%)	0 (0%)
Mesothelium	105 (1.3%)	88 (2.0%)	17 (0.5%)	0 (0%)	0 (0%)
Nervous system	93 (1.2%)	68 (1.6%)	21 (0.6%)	4 (3.3%)	0 (0%)
Blood	25 (0.3%)	20 (0.5%)	4 (0.1%)	1 (0.8%)	0 (0%)
Eyes	10 (0.1%)	8 (0.2%)	1 (0.1%)	1 (0.8%)	0 (0%)
Bone	9 (0.1%)	6 (0.1%)	3 (0.1%)	0 (0%)	0 (0%)
Unspecified	682 (8.6%)	461 (10.6%)	166 (4.8%)	46 (37.4%)	9 (32.1%)
Others	65 (0.8%)	31 (0.7%)	33 (1.0%)	0 (0%)	1 (3.6%)
Missing	330 (4.1%)	8 (0.2%)	321 (9.2%)	1 (0.8%)	0 (0%)

### 3.3 Number and Signal Analysis of Neurological Adverse Events for PD‐1 Inhibitors

**FIGURE 3:**
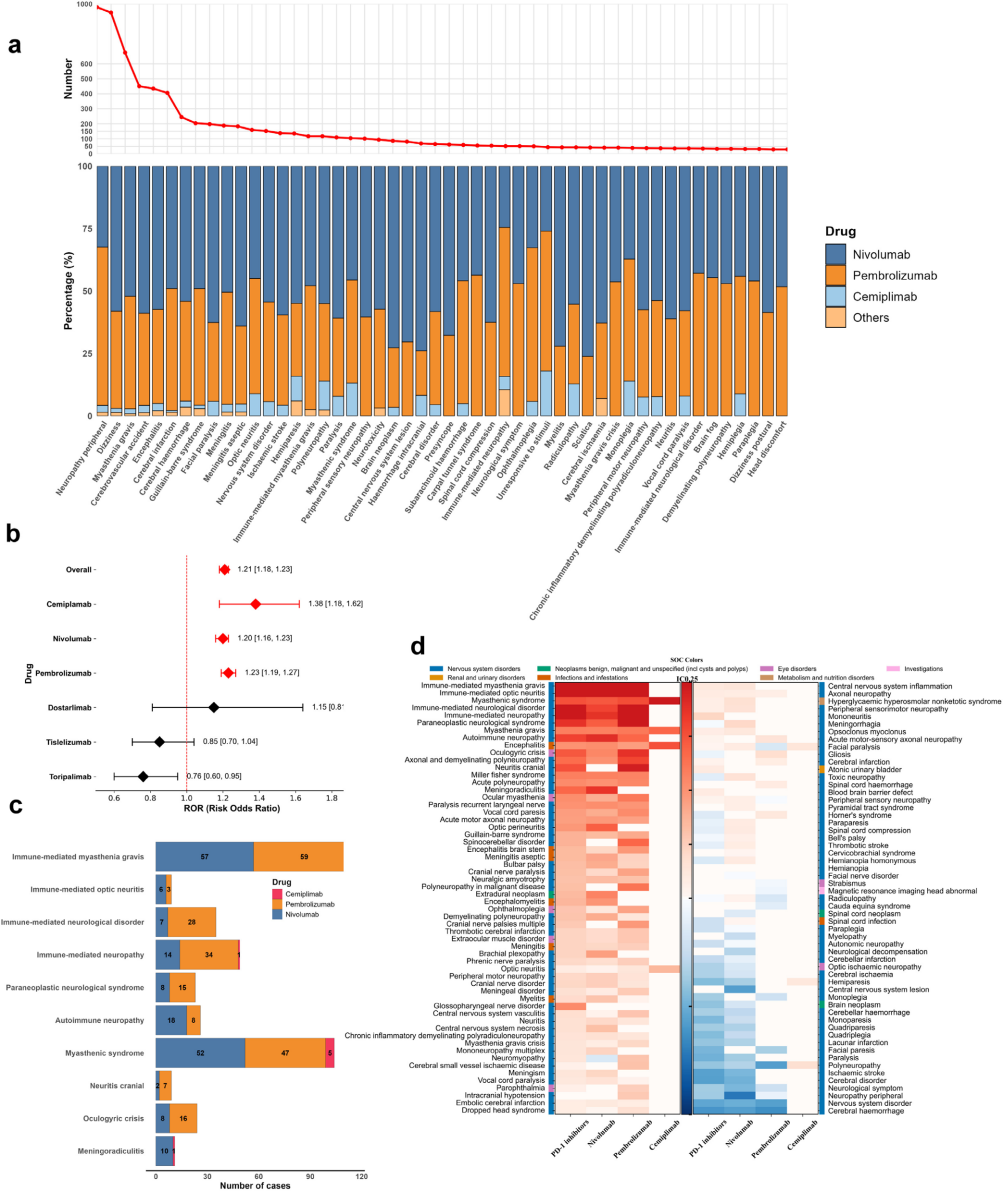
Analysis of neurological adverse events (nAEs) frequency and signal detection for PD‐1 inhibitors. (a) Upper panel: Line chart of frequency distribution for the top 50 most reported nAEs, ranked by occurrence. Lower panel: Stacked bar chart of proportional distribution across PD‐1 inhibitor types (nivolumab, pembrolizumab, cemiplimab, and others, including tislelizumab, dostarlimab, and toripalimab). (b) Forest plot of Reporting Odds Ratios (ROR) with 95% confidence intervals for neurological adverse events by individual PD‐1 inhibitors and overall PD‐1 inhibitor class. Diamond symbols indicate point estimates. (c) Stacked bar chart of case numbers for the top 10 strongest nAE signals associated with PD‐1 inhibitors, stratified by the three inhibitors (nivolumab, pembrolizumab, cemiplimab) with significant associations. (d) Heatmap of IC025 values for 115 nAEs with significant ROR signals across three PD‐1 inhibitors. Empty cells indicate < 3 cases or no reported events. Color intensity represents signal strength: Deeper red indicates stronger positive signals, blue indicates nonsignificant negative values.

In this study, we identified 362 neurological adverse event Preferred Terms (nPTs) induced by PD‐1 inhibitors from the FAERS database spanning Q4 2014 to Q2 2024 (Table S1). Within the analyzed PD‐1 inhibitors, nivolumab and pembrolizumab emerged as the predominant agents associated with nPTs, accounting for an average of 98.1% of cases across the top 50 most frequently reported PTs, whereas cemiplimab accounted for 1.6%. The most common nPTs of PD‐1 inhibitors were peripheral neuropathy(*N* = 978, 10.7%), dizziness (*N* = 942, 10.3%), and myasthenia gravis (*N* = 675, 7.4%) (Figure 3a).

**FIGURE 4:**
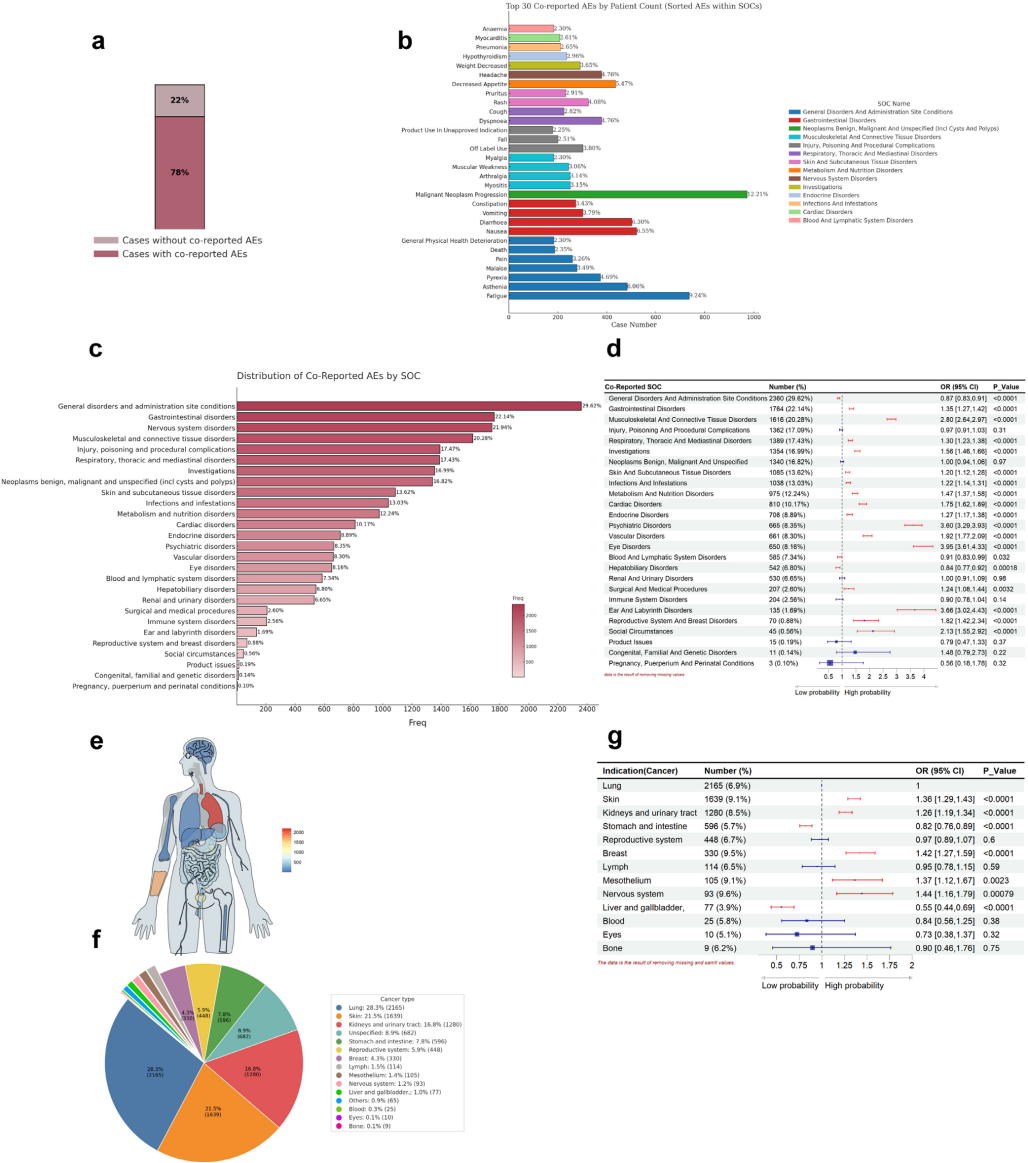
Distribution of co‐reported adverse events and indications, and their impact on nAEs induced by PD‐1 inhibitors. (a) The bar chart displays the co‐reported adverse event rates in patients with nAEs induced by PD‐1 inhibitors. (b) The proportion of occurrence of the top 30 most frequent co‐reported adverse events PTs among individuals with PD‐1 inhibitor–induced nAEs. The different colors of the bars represent the different SOCs to which these PTs belong. (c) The bar chart displays the frequency and proportion of co‐reported adverse events at the SOC level in the population with PD‐1 inhibitor–induced nAEs. The darker the color, the higher the frequency and proportion. (d) Logistic regression: Impact of co‐reported adverse events by System Organ Class (SOC) on PD‐1 inhibitor–induced nAEs. (e) The organ heatmap shows the distribution of tumors in different organs among patients with PD‐1 inhibitor–induced nAEs. (f) The pie chart presents the distribution of indications (types of tumors) in patients with PD‐1 inhibitor–induced nAEs. (g) Logistic regression forest plot: The impact of different indications (types of tumors) on PD‐1 inhibitor–induced nAEs.

Our signal analysis demonstrated that PD‐1 inhibitors (ROR: 1.21, 95% CI: 1.18–1.23) were significantly associated with nAEs. Stratified analysis of individual PD‐1 inhibitors revealed that nivolumab (ROR:1.20, 95% CI: 1.16–1.23), pembrolizumab (ROR: 1.23, 95% CI: 1.19–1.27), and cemiplimab (ROR: 1.38, 95% CI: 1.18–1.62) were significantly associated with nAEs, with cemiplimab exhibiting the strongest signal (Figure 3b). To further investigate specific nPTs signals, we adopted stricter criteria: both significant ROR and IC values. Figure 4d illustrates the IC 95% lower limit values for three significant PD‐1 inhibitors and the entire PD‐1 class for nPTs with ROR 95% lower limit values exceeding 1. For overall PD‐1 inhibitors, we identified 69 positive signals (IC025 range:0.09–4.91). Among the top 10 strongest PT signals, immune‐mediated myasthenia gravis (IC025: 4.91, *N* = 116) and myasthenic syndrome (IC025: 3.03, *N* = 104) emerged as the most frequently reported nPTs associated with PD‐1 inhibitors. Neuritis cranial(IC025:3.01) demonstrated a strong signal, despite its rare occurrence(*N* = 9) (Figure 3c,d). Figure S1 illustrates the MedDRA hierarchical relationships of the top 10 nPTs most strongly associated with PD‐1 inhibitors, with their primary SOC being nervous system disorders. Among specific PD‐1 inhibitors, Nivolumab and Pembrolizumab were associated with more PT signals, with 68 (IC025 range: 0.03–5.1) and 56 (IC025 range: 0.02–5.52) signals, respectively. The strongest signals for nivolumab were immune‐mediated optic neuritis (IC025: 5.1) and immune‐mediated myasthenia gravis (IC025: 4.76), while pembrolizumab showed peak signals in immune‐mediated neurological disorder (IC025: 5.52) and immune‐mediated myasthenia gravis (IC025: 5.13). Compared to the aforementioned agents, cemiplimab exhibited only 7 signals, with myasthenic syndrome yielding the highest signal (IC025: 4.72) and hemiparesis the lowest (IC025: 0.44). Notably, myasthenic syndrome, encephalitis, myasthenia gravis, and optic neuritis showed strong associations with all three PD‐1 inhibitors. Each PD‐1 inhibitor demonstrated unique neurological signals: meningoradiculitis associated with nivolumab (IC025: 3.44), cranial neuritis related to pembrolizumab (IC025: 3.85), and polyneuropathy correlated with cemiplimab (IC025: 0.71) (Figure 3d).

*E*‐value analysis indicated robust resistance to potential unmeasured confounding (Table S2). For PD‐1 inhibitors overall, an unmeasured confounder would need to be associated with both drug exposure and neurological adverse events by a risk ratio of 2.13‐fold each to explain away the observed lower confidence limit. Individual agents showed varying degrees of robustness: pembrolizumab (*E*‐value = 3.08, lower CI = 2.66), cemiplimab (*E*‐value = 2.94, lower CI = 2.17), and nivolumab (*E*‐value = 2.10, lower CI = 1.99). These values suggest that moderately strong unmeasured confounding would be required to fully account for the observed associations.

### 3.4 Sensitivity Analysis and Assessment of Unmeasured Confounding

To assess the robustness of associations between PD‐1 inhibitors and neurological adverse events, we conducted multivariable logistic regression controlling for measured confounders and computed *E*‐values to quantify vulnerability to unmeasured confounding. The *E*‐value represents the minimum strength of association an unmeasured confounder would need with both exposure and outcome to nullify an observed effect.

Sequential adjustment models were implemented (Table 2). Model 1 adjusted for reporter type, geographic region, and concomitant medication use. All associations remained significant: aRORs ranged from 1.20 (95% CI: 1.15–1.24) for nivolumab to 1.53 (95% CI: 1.35–1.74) for pembrolizumab, with lower‐bound E‐values from 1.57 to 2.04. Model 2 added patient age, sex, and body weight, yielding attenuated but persistent associations for the overall PD‐1 inhibitor class (aROR = 1.14, 95% CI: 1.07–1.22), nivolumab (aROR = 1.13, 95% CI: 1.05–1.20), and pembrolizumab (aROR = 1.35, 95% CI: 1.05–1.73). Cemiplimab could not be reliably estimated in Model 2 due to limited complete‐case data (*n* = 33). The persistence of associations across progressive adjustments, with *E*‐values indicating moderate to strong robustness to unmeasured confounding, supports the observed neurological safety signals. While unmeasured confounding of substantial magnitude cannot be excluded, the consistency of findings across analytic models suggests these associations are unlikely to be fully explained by uncontrolled bias.

**TABLE 2:** Sensitivity analysis: adjusted reporting odds ratios and e‐values for neurological adverse events associated with PD‐1 inhibitors.

Types of drug	Model	Case number with nAEs		aROR (95% CI)			*p*
		(Yes/No)			*E*‐value	*E*‐value	
		With PD‐1 inhibitors	Without PD‐1 inhibitors		(point)	(Lower CI)	
Total PD‐1 inhibitors	Model 1^a^	3,512/56,718	477,918/12,133,509	1.22 (1.18–1.26)	1.71	1.64	< 0.001
	Model 2^b^	1,006/13,360	102,163/1,775,415	1.14 (1.07–1.22)	1.54	1.34	< 0.001
Nivolumab	Model 1^a^	3,179/52,934	478,251/12,137,293	1.20 (1.15–1.24)	1.69	1.57	< 0.001
	Model 2^b^	935/12,562	102,234/1,776,213	1.13 (1.05–1.20)	1.51	1.28	< 0.001
Pembrolizumab	Model 1^a^	263/2,716	481,167/12,187,511	1.53 (1.35–1.74)	2.43	2.04	< 0.001
	Model 2^b^	69/767	103,100/1,788,008	1.35 (1.05–1.73)	2.04	1.28	0.018
Cemiplimab	Model 1^a^	70/1,068	481,360/12,189,159	1.53 (1.20–1.95)	2.43	1.69	< 0.001
	Model 2^b^	2/31	103,167/1,788,744	1.19 (0.28–5.01)	NA	NA	0.810

^a^ Model 1: Adjusted for reporter type (professional vs. unprofessional), geographic region (United States vs. other countries), and concomitant medication use (monotherapy vs. polypharmacy).

^b^ Model 2: Adjusted for reporter type, geographic region, concomitant medication use, age (continuous variable), sex (male vs. female), and body weight (continuous variable).

### 3.5 Influencing Factors for PD‐1 Inhibitor–Induced pAEs

We analyzed co‐reported adverse events as potential factors affecting nAEs of PD‐1 inhibitors. Among the 7968 PD‐1 inhibitor–related nAEs, 78% were accompanied by other adverse events, whereas 22% occurred in isolation (Figure 4a). At the PT level, the most frequently co‐reported AEs were malignant neoplasm progression (*N* = 973, 12.21%), fatigue (*N* = 736, 9.24%), and nausea (*N* = 522, 6.55%) (Figure 4b). Detailed frequencies and case percentages of co‐reported PT are presented in Table S3. At the SOC level, besides nervous system disorders (≥ 2 cases), general disorders and administration site conditions (*N* = 2360, 29.62%), gastrointestinal disorders (*N* = 1764, 22.14%), and musculoskeletal and connective tissue disorders (*N* = 1616, 20.28%) were the top 3 reported co‐reported AEs, appearing in over 20% of PD‐1 inhibitors‐induced nAes (Figure 4c).

Using univariate logistic regression analysis, we found that most co‐reported SOCs were significant influencing factors for PD‐1 inhibitor–related nAEs. For example, patients with eye disorders had 3.95 (OR: 3.95 [3.61, 4.33], *p* < 0.0001) times higher odds of developing neurologic adverse events following PD‐1 inhibitor treatment compared to those without eye disorders. Similarly, patients with ear and labyrinth disorders or psychiatric disorders had 3.66 (OR: 3.66 [3.02,4.43], *p* < 0.0001) and 3.60 (OR: 3.60 [3.29,3.93], *p* < 0.0001) times higher odds, respectively. In contrast, patients with hepatobiliary disorders had only 0.84 times the odds of developing nAEs compared to those without this condition (Figure 4d).

We further evaluated the associations between patient clinical characteristics (including sex, age, indications, and additional therapy) and PD‐1 inhibitor–associated nAEs. Stratified analyses demonstrated distinct risk patterns for nAEs across different indications. Significantly elevated nAE risks were found in patients with skin cancer (OR: 1.36 [1.29–1.43], *p* < 0.0001), kidneys and urinary tract cancer (OR: 1.26 [1.19–1.34], *p* < 0.0001), breast cancer (OR: 1.42 [1.27–1.59], *p* < 0.0001), mesothelium cancer (OR: 1.37 [1.12–1.67], *p* = 0.0023), and nervous system cancer (OR: 1.44 [1.16–1.79], *p* = 0.00079). Conversely, significantly reduced nAE risks were found in patients with stomach and intestine cancer (OR: 0.82 [0.76–0.89], *p* < 0.0001) and liver and gallbladder cancer (OR: 0.55 [0.44–0.69], *p* < 0.0001) (Figure 4g). Female patients demonstrated a marginally higher risk of nAEs compared with male patients (OR: 1.04 [1.01–1.08], *p* = 0.025). Compared with patients aged 45–65 years, those under 45 years showed a lower risk of nAEs (OR: 0.88 [0.80–0.97], *p* = 0.0081), while patients over 65 years exhibited a higher risk (OR: 1.10 [1.06–1.14], *p* < 0.0001). Lower risks of nAEs were identified in patients with body weight < 50 kg (OR: 0.76 [0.66, 0.86], *p* < 0.0001), reports from unprofessional submitters (OR: 0.86 [0.82, 0.90], *p* < 0.0001), cases from the United States (OR: 0.73 [0.70, 0.76], *p* < 0.0001), nivolumab treatment (OR: 0.89 [0.86, 0.91], *p* < 0.0001), and concurrent use of metabolic antagonists (OR: 0.89 [0.80, 0.99], *p* = 0.030). Higher risks of nAEs were observed in cases from Germany (OR: 1.26 [1.14–1.39], *p* < 0.0001), events occurring within 30 days of treatment initiation (OR: 1.13 [1.07–1.20], *p* < 0.0001), and concurrent administration of antineoplastic agents, including alkylating agents (OR: 1.42 [1.17–1.73], *p* = 0.00043), natural alkaloids and derivatives (OR: 1.48 [1.35–1.62], *p* < 0.0001), antitumor antibiotics (OR: 1.46 [1.17–1.83], *p* = 0.00099), and platinum‐based compounds (OR: 1.16 [1.08–1.25], *p* < 0.0001) (Figure S2).

### 3.6 Time to Onset Analysis for PD‐1 Inhibitor–Induced nAEs

**FIGURE 5:**
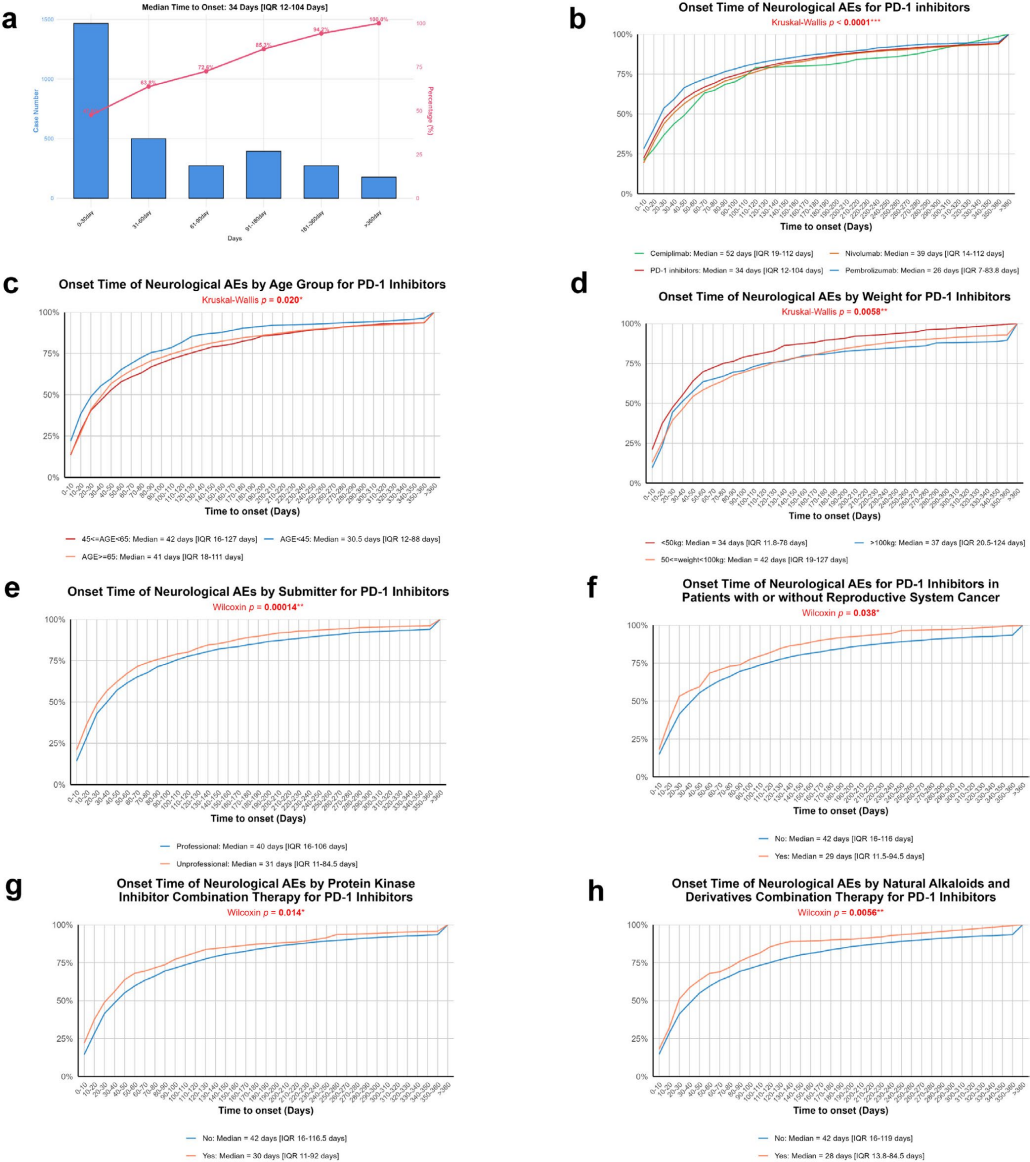
Analysis of factors influencing onset time of PD‐1 inhibitor–induced neurological adverse events (nAEs). (a) Histogram of onset time distribution for PD‐1 inhibitor–induced nAEs with cumulative incidence curve showing the proportion of events occurring over time (median onset: 34 days, IQR 12–154 days). (b‐h) Cumulative incidence curves comparing onset patterns across subgroups: (b) PD‐1 inhibitor types (cemiplimab, nivolumab, pembrolizumab, overall PD‐1 inhibitors), (c) age groups (45 ≤ AGE < 65, AGE < 45, AGE ≥ 65), (d) weight categories (< 50 kg, 50 ≤ weight < 100 kg, ≥ 100 kg), (e) reporter types (professional vs. nonprofessional), (f) reproductive system cancer status (with vs. without), (g) protein kinase inhibitor combination therapy (yes vs. no), and (h) natural alkaloids combination therapy (yes vs. no). Each panel displays median onset times with interquartile ranges and statistical test *p*‐values (Wilcoxon test for two‐group comparisons, Kruskal–Wallis test for multiple‐group comparisons).

Most nAEs (63.8%) occurred within 2 months of PD‐1 inhibitor initiation, with a median onset time of 34 days (IQR: 12–104 days). A small proportion of cases (5.2%) developed after 1 year (Figure 5a). The median onset times of nAEs varied significantly among PD‐1 inhibitors (*p* < 0.0001), ranging from 26 days (IQR: 7–83.8) for pembrolizumab to 52 days (IQR: 19–112) for cemiplimab(Figure 5b). Both age (*p* = 0.020) and body weight (*p* = 0.0058) significantly influenced nAE onset times. Patients aged < 45 years had earlier onset (median: 30.5 days; IQR: 12–88 days), while those with body weight 50–100 kg showed later onset (median: 42 days; IQR: 19–127 days) (Figure 5c,d). Unprofessional individuals showed significantly earlier onset than professionals (31 vs. 40 days; *p* = 0.00014) (Figure 5e). Significant associations with nAE onset were also found in reproductive system cancer (*p* = 0.038), concurrent use of protein kinase inhibitors (*p* = 0.014), and natural alkaloids and derivatives (*p* = 0.0056) (Figure 5f–h). Figure S3 exhibits the group with no significant variation in the time to onset.

### 3.7 Risk Factors Associated With Serious Outcomes of PD‐1 Inhibitor–Related nAEs

**FIGURE 6:**
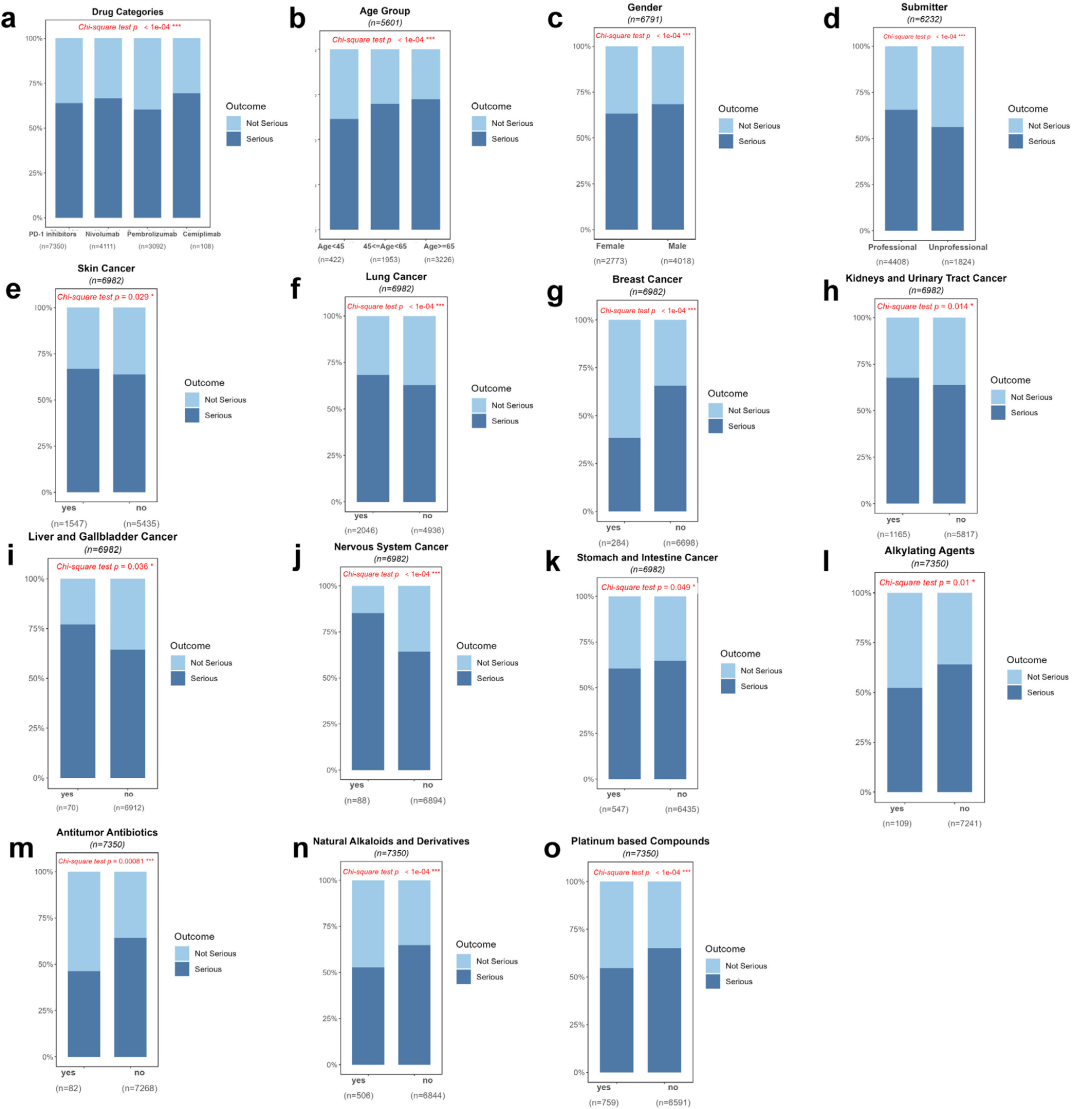
Analysis of factors influencing serious outcomes of PD‐1 inhibitor–induced neurological adverse events (nAEs). (a–o) Stacked bar charts showing proportions of serious vs. nonserious outcomes across subgroups: (a) PD‐1 inhibitor types (nivolumab, pembrolizumab, cemiplimab), (b) age groups (AGE < 45, 45 ≤ AGE < 65, AGE ≥ 65), (c) gender (female vs. male), (d) reporter types (professional vs. nonprofessional), (e) skin cancer, (f) lung cancer, (g) breast cancer, (h) kidney and urinary tract cancer, (i) liver and gallbladder cancer, (j) nervous system cancer, (k) stomach and intestine cancer, (l) alkylating agents combination, (m) antitumor antibiotics combination, (n) natural alkaloids and derivatives combination, and (o) platinum‐based compounds combination. Sample sizes and chi‐square test *p*‐values are shown for each comparison.

Among patients with documented outcomes who developed PD‐1 inhibitor–associated nAEs, 59% experienced serious outcomes, underscoring the importance of identifying risk factors for patient management optimization. The proportion of serious outcomes differed significantly between PD‐1 inhibitors (*p* < 0.0001), with cemiplimab showing the highest rate (69.4%) and pembrolizumab the lowest (60.3%) (Figure 6a). Advanced age was significantly associated with serious outcomes (*p* < 0.0001), and male patients demonstrated a higher incidence of serious outcomes compared to females (*p* < 0.0001) (Figure 6b,c). Healthcare professional‐reported cases demonstrated a significantly higher proportion of serious outcomes compared to unprofessional‐reported cases (*p* < 0.0001) (Figure 6d). Several cancer indications were identified as risk factors for serious outcomes from PD‐1 inhibitor–induced nAEs: skin cancer (*p* = 0.029), lung cancer (*p* < 0.0001), kidneys and urinary tract cancer (*p* = 0.014), liver and gallbladder cancer (*p* = 0.036), and nervous system cancer (*p* < 0.0001). We identified multiple cancer types associated with an upper risk of serious PD‐1 inhibitor–induced nAEs: skin cancer (*p* = 0.029), lung cancer (*p* < 0.0001), kidneys and urinary tract cancer (*p* = 0.014), liver and gallbladder cancer (*p* = 0.036), and nervous system cancer (*p* < 0.0001). Conversely, breast cancer (*p* < 0.0001) and gastrointestinal cancer (*p* = 0.049) were associated with a lower risk of serious outcomes (Figure 6e–k). Concurrent administration of four chemotherapeutic classes was associated with a lower risk of serious outcomes: alkylating agents (*p* = 0.01), antitumor antibiotics (*p* = 0.00081), natural alkaloids and derivatives (*p* < 0.0001), and platinum‐based compounds (*p* < 0.0001) (Figure 6l–o). Figure S4 shows the group with no significant difference in serious outcomes.

### 3.8 Temporal Trend Analysis

To further explore temporal trends in PD‐1 inhibitor–associated neurological adverse events (nAEs), we performed linear regression analysis using quarterly aggregated counts of nAEs from 2014 to 2024. As shown in Figure S5, the regression model demonstrated a statistically significant upward trend (*β* = 9.72 cases per quarter, 95% CI: 4.23–15.22, *p* = 0.001, *R* ^2^ = 0.242), indicating an average annual increase of approximately 39 nAE cases throughout the study period. This temporal analysis confirms a genuine increase in nAE reporting patterns over the decade‐long observation period.

## 4 Discussion

While previous studies have documented neurological adverse reactions (nAEs) associated with PD‐1 inhibitors, a large‐scale, systematic investigation of nAEs across the complete spectrum of PD‐1 inhibitors remains to be conducted. In this pharmacovigilance study, we present the first comprehensive analysis of PD‐1 inhibitor–induced neurological adverse reactions via real‐world data from the FAERS database. Through rigorous disproportionality analysis of the entire FAERS database, we systematically identified PD‐1 inhibitor–associated neurological adverse events at both aggregate and subcategory levels, while thoroughly examining their clinical manifestations and potential risk factors.

Following FDA approval of PD‐1 inhibitors for multiple indications, including melanoma and non‐small‐cell lung cancer, reports of immune‐related adverse events (irAEs) have shown a consistent upward trend. Notably, nAEs, although constituting only 6.93% of all PD‐1 inhibitor–related irAEs in the FAERS database, demonstrated a significant increase from 4.96% in 2014 to 7.67% in 2024. This substantial increase not only reflects the growing recognition of neurological risks associated with PD‐1 inhibitors but also emphasizes the absolute necessity for enhanced surveillance and management strategies in clinical settings. This observed trend can be attributed to several key factors: the growing usage of PD‐1 inhibitors in conjunction with other drugs, enhanced adverse event reporting awareness, the expansion of indications for which PD‐1 inhibitors are applicable, and the application of new types of PD‐1 inhibitors [1, 30]. Moreover, as documented in Anna Pous's case report, modifications in PD‐1 inhibitors protocols necessitated by the COVID‐19 pandemic may have contributed to the upsurge of neurological adverse events [31]. In line with previous research, our findings also demonstrated that pembrolizumab had a higher proportion of nAEs (7.44%) compared to other PD‐1 inhibitors. This elevated incidence may be attributed to pembrolizumab's dominant market position, broader clinical application, and enhanced adverse event reporting due to greater familiarity among healthcare providers [32]. This trend underscores the critical need for clinicians to recognize the rising neurological risks associated with PD‐1 inhibitors, particularly pembrolizumab, as their widespread use could lead to more frequent and severe adverse events, necessitating a reevaluation of safety protocols in clinical practice.

Among the neurological adverse events (nAEs) reported with PD‐1 inhibitors, peripheral neuropathy, dizziness, myasthenia gravis, cerebrovascular accidents, and encephalitis were the most frequent. Notably, myasthenia gravis and encephalitis showed significant associations, highlighting the risk of serious neurological toxicity, consistent with prior findings [33]. Signal analysis identified immune‐mediated myasthenia gravis and myasthenic syndrome among the top 10 nAEs, with high frequency and strong signal strength. These events, marked by muscle weakness, align with previously reported neurological toxicities [34]. Two key mechanisms may underlie these events: hidden autoimmunity, where PD‐1 blockade disrupts immune tolerance, activating pre‐existing autoimmune responses; and molecular mimicry, where structural similarities between tumor antigens and neuromuscular junction proteins trigger T‐cell cross‐reactivity and neuromuscular damage [35]. Myasthenic syndrome was strongly linked to pembrolizumab, nivolumab, and cemiplimab, emphasizing the necessity for diligent monitoring of muscle‐related nAEs in patients receiving these PD‐1 inhibitors.

Our study found that neuritis cranial, meningoradiculitis, encephalitis, and encephalitis brain stem were significantly associated with PD‐1 inhibitors, exhibiting strong signals. These central nervous system (CNS)‐related neurological adverse events (nAEs), though serious, are mostly treatable. One study reported that PD‐1 inhibitor–induced encephalitis responded well to immunosuppressive therapy, with most patients achieving good prognoses [36]. This underscores the importance of early diagnosis and timely intervention to improve outcomes. Additionally, we identified nAEs uniquely linked to specific PD‐1 inhibitors. Polyneuropathy in malignant disease and multiple cranial nerve palsies were associated only with pembrolizumab, facial paralysis with cemiplimab, and brachial plexopathy with nivolumab. While PD‐1 inhibitors share neurotoxicity risks, specific agents may have distinct profiles. Existing literature views nAEs as a class effect, with little research on drug‐specific variations [37]. Further research is required to confirm these connections and assess whether differences arise from drug properties, immune modulation, or patient factors, aiding in targeted monitoring and personalized treatment.

The observed heterogeneity in neurological adverse events across different PD‐1 inhibitors and cancer types reflects complex molecular mechanisms involving tumor microenvironment diversity and immune regulatory dysfunction. Recent advances have demonstrated that tumor microenvironments exhibit profound heterogeneity even within histologically similar subtypes, characterized by distinct immune infiltration patterns, cytokine networks, and antigen presentation capabilities [38, 39]. The differential nAE risks we observed among various cancer types—with elevated rates in skin, kidney, and nervous system malignancies—likely reflect these distinct inflammatory landscapes that create varying propensities for cross‐reactive immune responses targeting neural antigens following PD‐1 blockade. Central to understanding PD‐1 inhibitor neurotoxicity is the dysregulation of TNF‐α signaling pathways, which operate at the crossroads of cellular survival and death through complex networks involving MAPK, Akt, and NF‐κB cascades [40]. When PD‐1 blockade restores T‐cell function, excessive TNF‐α production may trigger neuroinflammatory cascades that ultimately manifest as encephalitis, myasthenia gravis, and cranial nerve disorders documented in our analysis.

Macrophage polarization represents another pivotal mechanism underlying PD‐1 inhibitor neurotoxicity. The delicate balance between pro‐inflammatory M1 and anti‐inflammatory M2 macrophage phenotypes becomes critically disrupted following immune checkpoint inhibition [41], promoting sustained M1 activation within the central nervous system. This dysregulated macrophage activation contributes to the persistent nature of neurological adverse events and accounts for the high rates of serious outcomes (59%) observed in our cohort. The differential capacity of specific PD‐1 inhibitors to modulate these immune regulatory networks may partially explain why cemiplimab demonstrated the strongest neurotoxicity signal in our analysis [42], suggesting the existence of drug‐specific mechanisms that extend beyond their shared PD‐1 blocking activity.

In studies on factors influencing neurological adverse events (nAEs) from PD‐1 inhibitors, we found that age and female sex increased nAE risk, while being underweight was associated with a reduced risk. The role of age in neurological toxicity remains debated. B. Fox's study found that patients over 80 were less likely to develop adverse events than those aged 75–79, suggesting a decline in immune reactivity [43]. However, Simone Rossi's study showed that most elderly patients developed chronic nAEs with poorer prognoses, emphasizing the need for long‐term monitoring [44]. Despite differences, our findings confirm that age is a key risk factor requiring careful assessment in elderly patients. Female patients had a slightly higher nAE risk, likely due to stronger immune responses, influenced by higher IFN‐α levels, TLR7 expression, and estrogen‐mediated immune modulation. In contrast, testosterone's immunosuppressive effects may lower nAE incidence in males [45]. Interestingly, underweight patients had a lower nAE risk, possibly due to reduced baseline immune activation and lower inflammatory cytokine levels, limiting excessive immune responses that drive PD‐1‐related neurotoxicity [46]. This underscores the necessity for clinicians to closely assess patient demographics and tailor monitoring strategies accordingly, particularly for high‐risk groups like elderly patients and women, to enhance safety during PD‐1 inhibitor therapy. Given these identified risk factors, clinicians should prioritize intensive neurological surveillance for vulnerable populations, particularly patients over 65 years, women, and those with skin, kidney, urinary tract, or nervous system cancers. A proactive monitoring approach involving baseline neurological evaluation followed by structured assessments every 2 weeks during the initial treatment phase is warranted for these patients. Early recognition of concerning symptoms—including progressive muscle weakness, persistent severe headaches, unexplained fever, or changes in mental status—should prompt immediate neurological consultation to prevent progression to more severe complications.

Our study found that cases reported by nonprofessionals were less likely to involve neurological adverse events (nAEs), likely due to underreporting or lack of awareness of neurological symptoms. Additionally, we observed significant country‐specific differences in the incidence of nAEs. For instance, the USA reported a lower incidence, while Germany had a higher incidence. A study by Katsuhara et al. found similar discrepancies, with Japan showing a lower correlation between SGLT2 inhibitors and acute renal failure, suggesting that reporting trends and prescription practices across countries affect adverse event incidence [47]. The type of PD‐1 inhibitor also plays a role in nAE occurrence. Nivolumab was associated with a lower risk of neurological toxicity compared to pembrolizumab, which is different from previous studies that showed similar incidence rates of nAEs for both drugs [48]. This discrepancy is noteworthy and requires further attention. In combination therapies, we found that platinum‐based compounds significantly increased the risk of nAEs, consistent with prior reports of platinum‐induced neurotoxicity [49, 50]. Additionally, antitumor antibiotics and alkylating agents also increased the likelihood of nAEs when combined with PD‐1 inhibitors, despite their individual neurotoxicity not being significant in earlier studies [51, 52, 53, 54]. This may be due to drug interactions with PD‐1 inhibitors, highlighting the need for clinicians to carefully consider patient profiles and drug combinations to minimize neurological adverse events.

In this study, we observed that PD‐1 inhibitor–associated neurological adverse events (nAEs) exhibited distinct patterns across different cancer types. We found that patients with skin, kidney, urinary tract, breast, mesothelial, and nervous system malignancies showed higher nAE incidence, whereas those with gastrointestinal and hepatobiliary cancers demonstrated lower rates. While previous studies have documented cancer type‐specific variations in immune‐related adverse events (irAEs) [11], our findings uniquely characterize the differential patterns of PD‐1 inhibitor–induced neurological toxicity across various cancer types. These observations have important clinical implications, suggesting the necessity for cancer type‐specific monitoring and management approaches. A potential mechanistic explanation for these variations may lie in the cancer type‐specific immune microenvironment differences, as demonstrated by Kennedy et al. [55]. Given these cancer type‐specific patterns, further research is needed to explore the underlying mechanisms driving these variations, particularly how different tumor microenvironments influence the development of PD‐1 inhibitor–related neurological toxicity.

Our analysis revealed that 78% of PD‐1 inhibitor–associated neurological adverse events (nAEs) co‐occurred with other adverse reactions, with most showing a positive correlation with nAE development. We observed hypothyroidism align with (an endocrine disorder) in approximately 3% of nAE cases, while gastrointestinal manifestations (diarrhea and nausea) occurred in over 6% of cases. These findings suggested mechanistic associations between neurological, gastrointestinal, and endocrine adverse events. These associations were previously found by Barron et al., who demonstrated that gastrointestinal and neurological manifestations represent predominant chronic immune‐related adverse events (irAEs) [56]. The underlying mechanisms likely involve interactions between the brain‐gut axis and gut microbiota. Gut microbiota‐derived metabolites, particularly short‐chain fatty acids (SCFAs), can modulate brain function through neuroendocrine signaling and immune regulation. Specifically, SCFAs such as butyrate can activate T cells, leading to enhanced immune responses. This altered immune function, combined with gut microbiota‐mediated modulation of blood–brain barrier permeability, may enhance neurological toxicity during PD‐1 inhibitor therapy by affecting central nervous system immune responses [57, 58, 59]. Future research should focus on understanding the mechanisms behind PD‐1 inhibitor–induced neurological toxicity, exploring the role of gut microbiota and immune responses, and identifying biomarkers to predict these adverse events and improve patient management.

The onset timing of PD‐1 inhibitor–associated nAEs showed notable differences from previous reports. While Fellner et al. reported a median onset of 56 days for immunotherapy‐induced neurological adverse events (nAEs) [60], we observed a considerably shorter median onset of 34 days. Another study reported 36 days for peripheral neurological toxicity [61], confirming the rapid occurrence of nAEs following PD‐1 inhibitor initiation and emphasizing the importance of early monitoring. We also observed drug‐specific differences: pembrolizumab had the shortest median onset of 26 days, 26 days earlier than cemiplimab. Supporting this concern, Charabi et al. reported a fatal case of longitudinal extensive transverse myelitis developing within 3 weeks of pembrolizumab initiation, emphasizing the critical need for vigilant early‐phase monitoring [62]. Additionally, we identified several risk factors associated with earlier nAE onset, including age < 45 years, body weight < 50 kg, reproductive system malignancies, and combination therapy with protein kinase inhibitors or natural alkaloids. These findings suggest the need for intensified monitoring in high‐risk populations. The distinct onset patterns observed across different PD‐1 inhibitors necessitate tailored surveillance strategies. Pembrolizumab's rapid median onset of 26 days calls for heightened vigilance during the first month of treatment, while cemiplimab's combination of delayed onset and highest severity rate warrants sustained monitoring throughout the initial treatment cycle. Additionally, the accelerated onset patterns seen in skin cancer and nervous system cancer patients underscore the need for more frequent neurological assessments in these populations during early treatment phases.

Among patients with PD‐1 inhibitor–associated neurological adverse events (nAEs), 64% experienced severe outcomes, including death, life‐threatening conditions, or prolonged hospitalization. These findings align with Smith et al.'s observations [63], where among 58 nAE cases, only 46.6% achieved complete remission, with the remainder experiencing fatal outcomes or permanent neurological deficits. Among different PD‐1 inhibitors, cemiplimab showed the highest severity rate (69.4%), while pembrolizumab demonstrated the lowest (60.3%), indicating the need for drug‐specific risk assessment. Elderly patients demonstrated increased severity of nAEs, consistent with previous reports of age‐related elevation in treatment discontinuation due to immune‐related adverse events [64]. Male patients exhibited significantly higher severity rates than females, potentially attributable to sex‐specific variations in immune responses. Severity rates varied markedly by cancer type, with elevated rates in cutaneous, pulmonary, renal, urological, hepatic, and neurological malignancies compared to breast and gastrointestinal cancers. Galvez et al. attributed these variations to tumor‐specific immune microenvironment characteristics, including differential inflammatory cytokine profiles and immune cell infiltration patterns [65]. Notably, concurrent administration of alkylating agents and platinum‐based compounds was associated with reduced severity rates, suggesting potential protective effects against PD‐1 inhibitor–induced toxicities.

The geographic distribution of neurological adverse event reports, with the United States and Japan contributing 56.9% of cases, likely reflects differences in pharmacovigilance infrastructure, clinical practice patterns, and regulatory reporting requirements across healthcare systems. Countries with established adverse event monitoring programs may show higher detection rates for neurological manifestations, while differences in reporting practices, healthcare provider experience, and regulatory standards across regions contribute to varied data collection. These international differences in pharmacovigilance systems may affect the observed patterns of PD‐1 inhibitor–associated neurological toxicity, highlighting the need for standardized global surveillance approaches to improve risk assessment across different populations and healthcare settings.

This study has several limitations that should be acknowledged while considering its contributions to understanding PD‐1 inhibitor safety profiles. As a pharmacovigilance study utilizing the FDA Adverse Event Reporting System (FAERS) database, our analysis leverages the strengths of large‐scale real‐world data but is inherently based on voluntary reporting, which may result in varying reporting rates across different healthcare settings and geographic regions. Neurological adverse events with subtle presentations might be underreported or attributed to disease progression, while highly publicized cases may receive increased reporting attention. Although disproportionality analysis provided valuable signal detection capabilities for this comprehensive safety assessment, it represents an exploratory approach that identifies associations rather than establishing definitive causal relationships between PD‐1 inhibitors and neurological adverse events. Our analysis was further limited by incomplete data for certain variables, including patient weight and onset timing, constraints in comprehensively controlling potential confounding variables such as genetic polymorphisms and prior therapeutic interventions, limited data on newer PD‐1 inhibitors like cemiplimab, and incomplete patient‐specific clinical data that restricted our ability to develop predictive models for identifying high‐risk patient populations. While acknowledging these inherent limitations of spontaneous reporting systems, it is important to note that pharmacovigilance studies remain essential for identifying potential safety signals, particularly for rare neurological events that may not be captured in clinical trials due to limited sample sizes and follow‐up periods. Given these limitations, future prospective studies should collect comprehensive clinical data to develop predictive models for identifying high‐risk patient populations, which would provide important clinical guidance for patient monitoring and management. Biomarker studies and mechanistic investigations are also needed to better understand the pathophysiology of PD‐1 inhibitor–induced neurotoxicity and identify susceptible patient populations. Future research should prioritize mechanistic studies to understand the pathophysiology of PD‐1 inhibitor neurotoxicity, develop validated biomarkers for early detection, and establish evidence‐based clinical guidelines for neurological monitoring and management in immunotherapy patients. Despite these limitations, this study provides important safety signals that warrant clinical attention and represents one of the largest systematic evaluations of PD‐1 inhibitor–associated neurological complications to date, offering valuable insights for clinical practice and regulatory decision‐making.

## 5 Conclusions

In conclusion, a pharmacovigilance analysis of nAEs associated with PD‐1 inhibitors via the FAERS database from 2014 to 2024 revealed significant trends and risk factors. The study demonstrated an increasing proportion of nAE reports, with nivolumab and pembrolizumab being most frequently reported, and cemiplimab exhibiting the strongest signal. Signal analysis identified central nervous system (CNS) events and myasthenia gravis as the most notable nAE signals. Furthermore, nivolumab showed a stronger signal for encephalitis, while pembrolizumab had a notable association with peripheral neuropathy. Cemiplimab's strongest signal was linked to myasthenia gravis. Risk factors encompassed patient demographics and treatment characteristics: elderly patients, females, and those with specific cancers (skin, kidney, urinary tract) were more susceptible. Combination therapies with platinum‐based compounds, antitumor antibiotics, and alkylating agents further increased nAE risks. Notably, PD‐1 inhibitor–specific variations were observed: pembrolizumab showed the shortest nAE onset time, particularly in younger, lower‐weight patients. Serious outcome rates differed among inhibitors, with cemiplimab highest and pembrolizumab lowest, with elderly and male patients experiencing higher rates. Based on these findings, we recommend enhanced neurological surveillance for high‐risk populations, including elderly patients, females, and those with skin, kidney/urinary tract, or nervous system cancers, with baseline assessment and regular monitoring during the first 2 months. Drug‐specific monitoring strategies are necessary: pembrolizumab requires more frequent early surveillance due to its rapid onset and risk of peripheral neuropathy; nivolumab necessitates ongoing monitoring for encephalitis throughout treatment; and cemiplimab warrants closer surveillance given its higher severity rate and association with myasthenic syndromes. Clinicians should evaluate progressive muscle weakness, severe headaches, altered mental status, or visual disturbances promptly and refer for immediate neurological consultation. When patients develop eye, ear, psychiatric, or endocrine disorders during treatment, clinicians should carefully monitor for neurological symptoms, as these events often co‐occur and may reflect systemic neurotoxicity. Future research should prioritize developing validated risk stratification tools, identifying biomarkers for early detection, and establishing standardized management protocols through collaboration between oncologists and neurologists. The study underscores the critical need for comprehensive risk management and highlights the importance of future research to validate findings and explore underlying mechanisms.

## Funding

This work was supported by grants from the National Natural Science Foundation of China (No. 82302332 and 82472696).

## Ethics Statement

The authors have nothing to report.

## Consent

All of the authors are aware of and agree to the content of the paper and their being listed as co‐authors of the paper.

## Conflicts of Interest

The authors declare no conflicts of interest.

## Supporting information


**Figure S1:** Sankey diagram showing the hierarchical relationships of the top 10 nAEs (based on signal strength with overall PD‐1 inhibitors) through MedDRA levels—from Preferred Terms (PT, the most specific level describing individual medical concepts), to High Level Terms (HLT, grouping related PTs), to High Level Group Terms (HLGT, broader grouping of related terms), and finally to System Organ Class (SOC, the highest level representing physiological systems).

**Figure S2:** Logistic Regression Forest Plot: Shows the impact of different clinical features and concomitant medications on PD‐1 inhibitor–induced nAEs. Includes clinical features such as gender, age, weight, and various concomitant medications.

**Figure S3:** Analysis of factors influencing the onset time of PD‐1 inhibitor–induced neurological adverse events (nAEs) showing no significant differences. (a‐m) Cumulative incidence curves comparing onset patterns across subgroups with nonsignificant differences: (a) alkylating agents combination therapy, (b) antitumor antibiotics combination therapy, (c) breast cancer status, (d) liver and gallbladder cancer status, (e) hormones and antihormones combination therapy, (f) kidney and urinary tract cancer status, (g) lung cancer status, (h) lymph cancer status, (i) metabolic antagonists combination therapy, (j) mesothelioma cancer status, (k) nervous system cancer status, (l) gender, and (m) outcome severity (serious vs. nonserious). Each panel displays median onset times with interquartile ranges and Wilcoxon test *p*‐values showing no statistically significant differences between groups.

**Figure S4:** Analysis of factors influencing serious outcomes of PD‐1 inhibitor–induced neurological adverse events (nAEs) showing no significant differences. (a–f) Stacked bar charts showing proportions of serious versus nonserious outcomes across subgroups with nonsignificant differences: (a) hormones and antihormones combination therapy, (b) lymph cancer status, (c) metabolic antagonists combination therapy, (d) weight categories (< 50 kg, 50 ≤ weight < 100 kg, ≥ 100 kg), (e) protein kinase inhibitor combination therapy, and (f) mesothelioma cancer status. Sample sizes and chi‐square test *p*‐values are shown for each comparison, demonstrating no statistically significant differences between groups.

**Figure S5:** Linear regression analysis of neurological adverse events (nAEs) temporal trend for PD‐1 inhibitors from Q4 2014 to Q2 2024. Scatter plot showing quarterly counts of neurological adverse events (blue crosses) with fitted linear regression line (red). The regression demonstrates a significant upward trend in nAE reporting over the 10‐year study period (*β* = 9.72 cases per quarter, 95% CI: 4.23–15.22, *p* = 0.001, *R* ^2^ = 0.242).

**Table S1:** The number of occurrences of each PT‐level neurological adverse event for each PD‐1 inhibitor.

**Table S2:** *E*‐values Assessing robustness to unmeasured confounding for primary associations between PD‐1 inhibitors and neurological adverse events.

**Table S3:** The proportion and number of PT‐level co‐reported adverse events among patients with PD‐1 inhibitor‐induced neurological adverse reactions.

## Data Availability

The datasets used in this study are accessible through the FDA Adverse Event Reporting System (FAERS), and any data analyzed during the study can be obtained from the corresponding author upon reasonable request.
