# Supplementary figures and images for: Multidimensional Assessment of Neurological Adverse Reactions Related to PD‐1 Inhibitors: A Real‐World Pharmacovigilance Study
Source: CNS Neurosci Ther. 2026 Jan 5;32(1):e70734. doi: 10.1002/cns.70734 (PMC12767002; doi:10.1002/cns.70734)

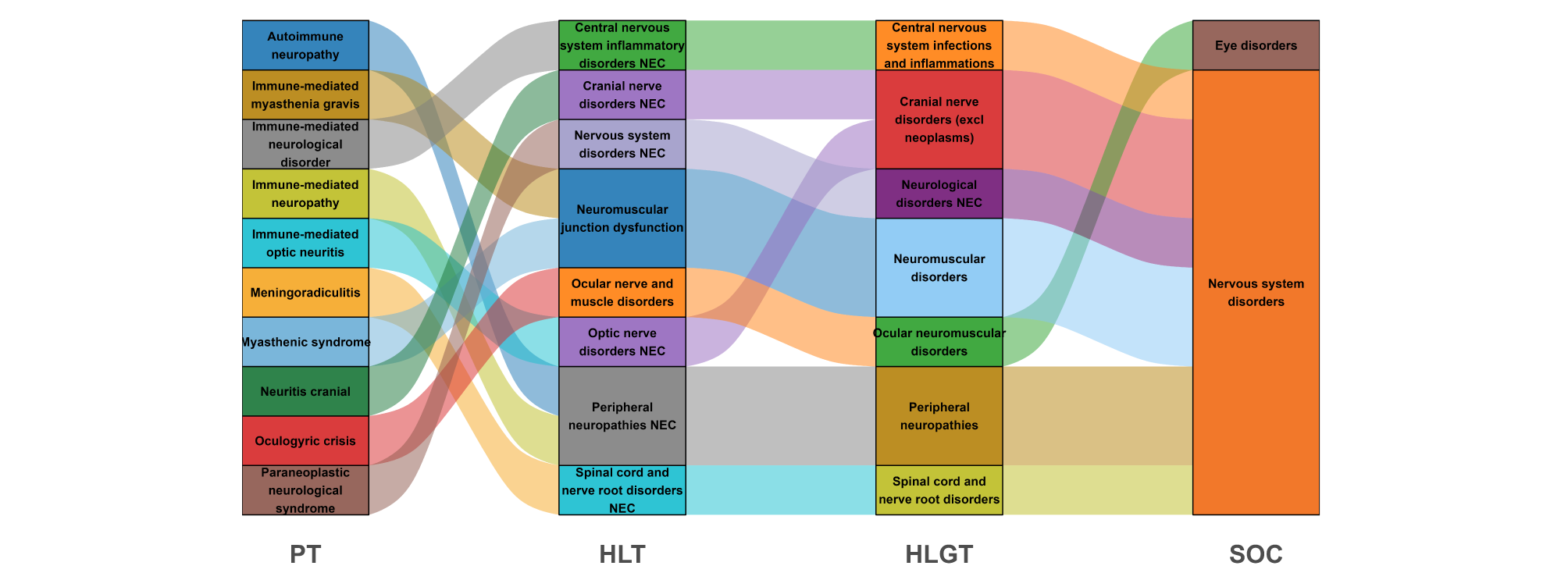

Supplement: Supplementary file 1 — Figure S1: Sankey diagram showing the hierarchical relationships of the top 10 nAEs (based on signal strength with overall PD‐1 inhibitors) through MedDRA levels—from Preferred Terms (PT, the most specific level describing individual medical concepts), to High Level Terms (HLT, grouping related PTs), to High Level Group Terms (HLGT, broader grouping of related terms), and finally to System Organ Class (SOC, the highest level representing physiological systems). [file CNS-32-e70734-s001.tif]

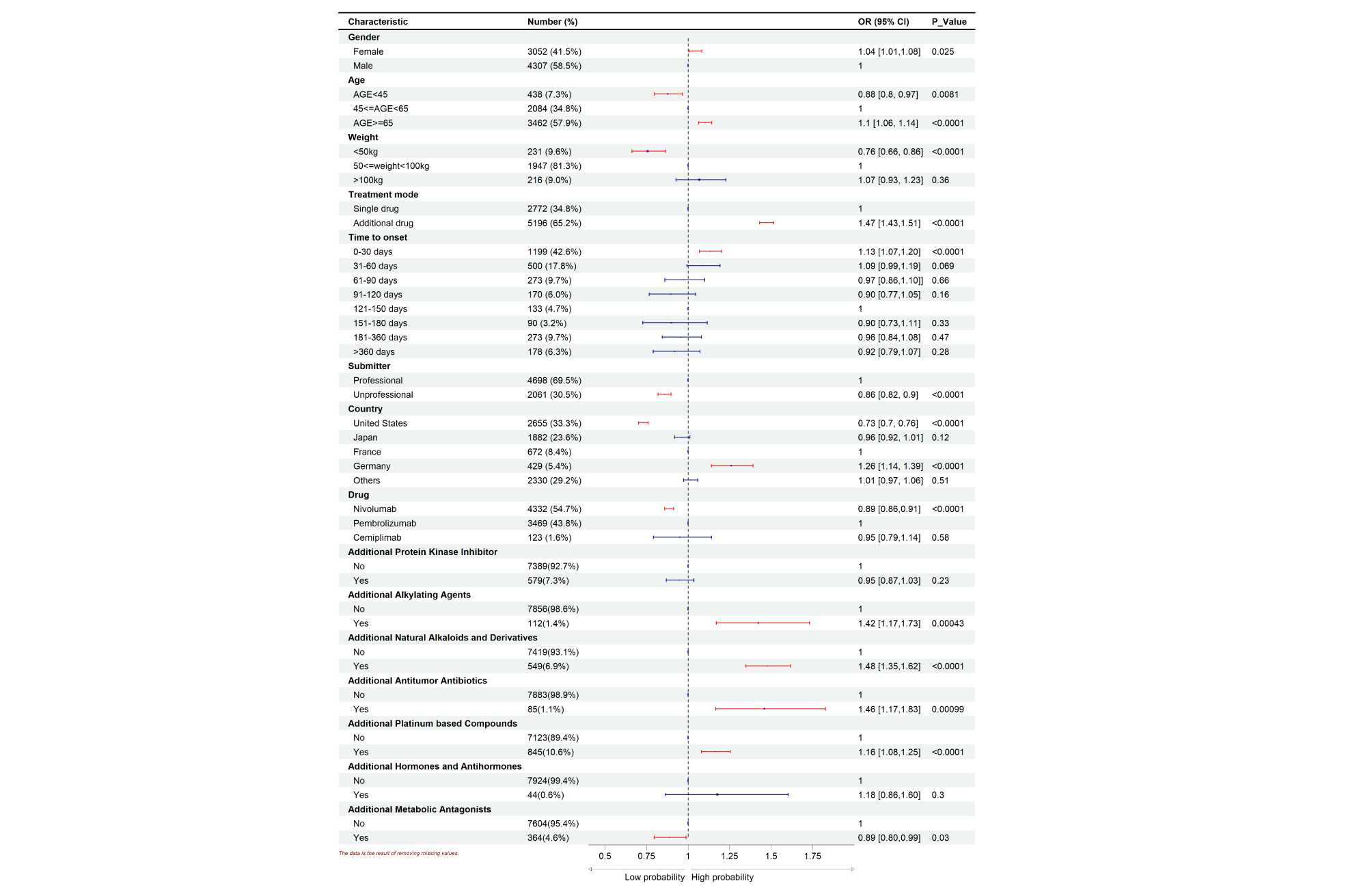

Supplement: Supplementary file 2 — Figure S2: Logistic Regression Forest Plot: Shows the impact of different clinical features and concomitant medications on PD‐1 inhibitor–induced nAEs. Includes clinical features such as gender, age, weight, and various concomitant medications. [file CNS-32-e70734-s002.tif]

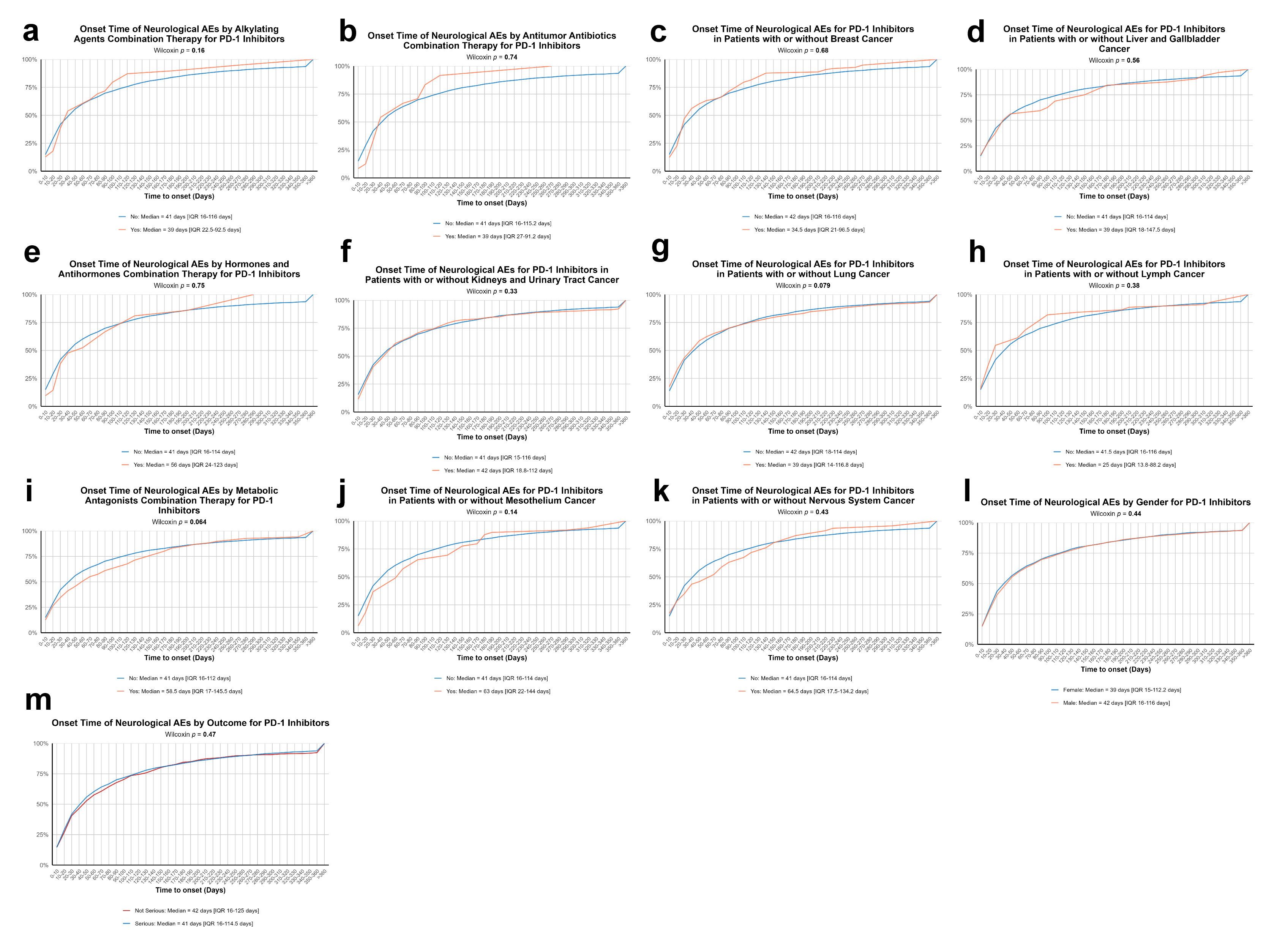

Supplement: Supplementary file 3 — Figure S3: Analysis of factors influencing the onset time of PD‐1 inhibitor–induced neurological adverse events (nAEs) showing no significant differences. (a‐m) Cumulative incidence curves comparing onset patterns across subgroups with nonsignificant differences: (a) alkylating agents combination therapy, (b) antitumor antibiotics combination therapy, (c) breast cancer status, (d) liver and gallbladder cancer status, (e) hormones and antihormones combination therapy, (f) kidney and urinary tract cancer status, (g) lung cancer status, (h) lymph cancer status, (i) metabolic antagonists combination therapy, (j) mesothelioma cancer status, (k) nervous system cancer status, (l) gender, and (m) outcome severity (serious vs. nonserious). Each panel displays median onset times with interquartile ranges and Wilcoxon test p‐values showing no statistically significant differences between groups. [file CNS-32-e70734-s004.tif]

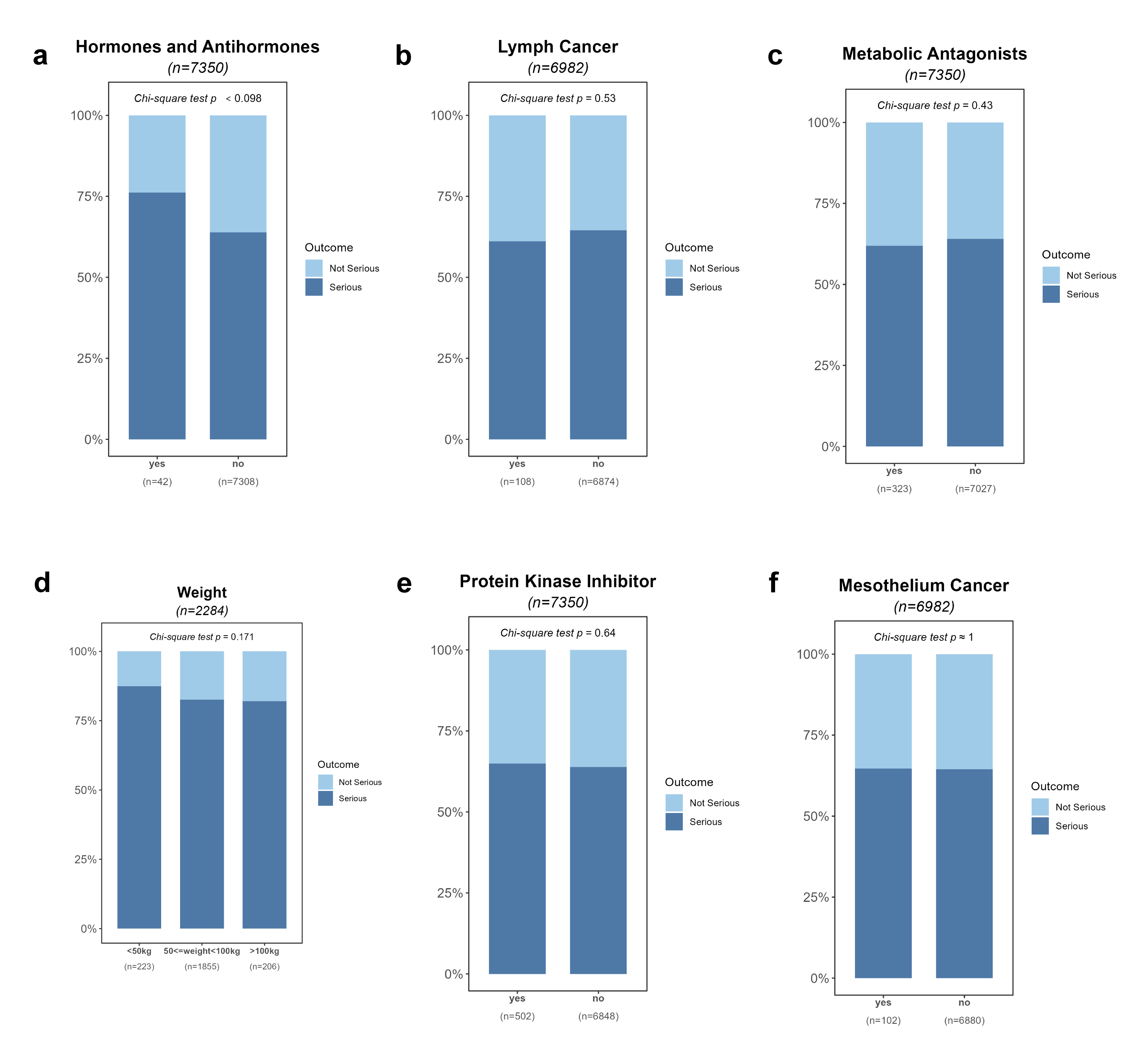

Supplement: Supplementary file 4 — Figure S4: Analysis of factors influencing serious outcomes of PD‐1 inhibitor–induced neurological adverse events (nAEs) showing no significant differences. (a–f) Stacked bar charts showing proportions of serious versus nonserious outcomes across subgroups with nonsignificant differences: (a) hormones and antihormones combination therapy, (b) lymph cancer status, (c) metabolic antagonists combination therapy, (d) weight categories (< 50 kg, 50 ≤ weight < 100 kg, ≥ 100 kg), (e) protein kinase inhibitor combination therapy, and (f) mesothelioma cancer status. Sample sizes and chi‐square test p‐values are shown for each comparison, demonstrating no statistically significant differences between groups. [file CNS-32-e70734-s006.tif]

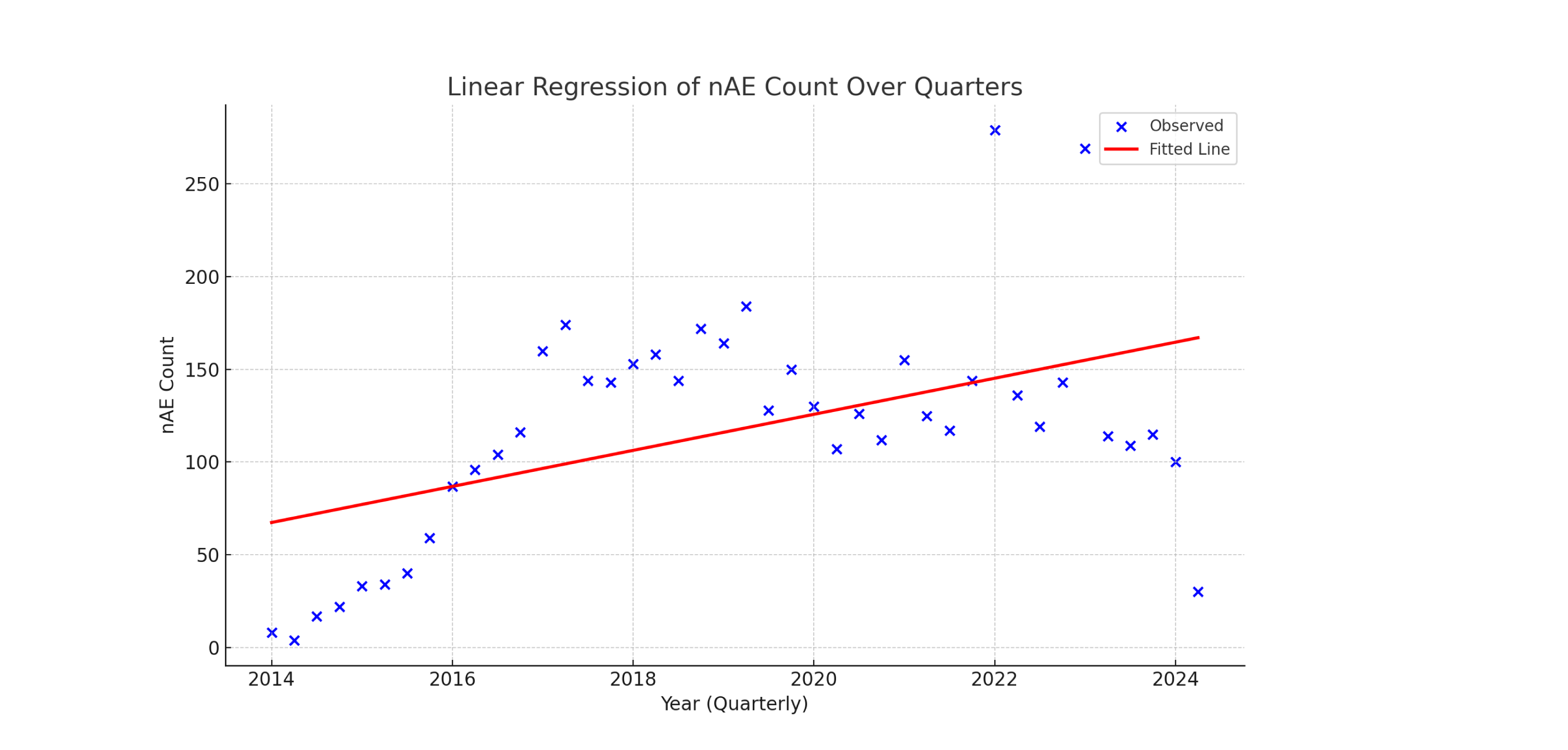

Supplement: Supplementary file 5 — Figure S5: Linear regression analysis of neurological adverse events (nAEs) temporal trend for PD‐1 inhibitors from Q4 2014 to Q2 2024. Scatter plot showing quarterly counts of neurological adverse events (blue crosses) with fitted linear regression line (red). The regression demonstrates a significant upward trend in nAE reporting over the 10‐year study period (β = 9.72 cases per quarter, 95% CI: 4.23–15.22, p = 0.001, R 2 = 0.242). [file CNS-32-e70734-s008.tif]
