# Supplementary material for: Multidimensional Assessment of Neurological Adverse Reactions Related to PD‐1 Inhibitors: A Real‐World Pharmacovigilance Study
Source: CNS Neurosci Ther. 2026 Jan 5;32(1):e70734. doi: 10.1002/cns.70734 (PMC12767002; doi:10.1002/cns.70734)
Supplement: Supplementary file 7 — Table S2: E‐values Assessing robustness to unmeasured confounding for primary associations between PD‐1 inhibitors and neurological adverse events. [file CNS-32-e70734-s005.docx]

| **Drug** | **ROR** | **Lower_CI** | **Upper_CI** | **E-value point** | **E-value Lower CI** |
| --- | --- | --- | --- | --- | --- |
| PD-1 inhibitors | 1.21 | 1.18 | 1.23 | 2.22 | 2.13 |
| Nivolumab | 1.20 | 1.16 | 1.23 | 2.10 | 1.99 |
| Pembrolizumab | 1.23 | 1.19 | 1.27 | 3.08 | 2.66 |
| Cemiplimab | 1.38 | 1.18 | 1.62 | 2.94 | 2.17 |
| **Table S2:** E-values Assessing Robustness to Unmeasured Confounding for Primary Associations Between PD-1 Inhibitors and Neurological Adverse Events | | | | | |
